# Pseudosterins A–C, Three 1-Ethyl-3-formyl-β-carbolines from *Pseudostellaria heterophylla* and Their Cardioprotective Effects

**DOI:** 10.3390/molecules26165045

**Published:** 2021-08-20

**Authors:** Guo-Bo Xu, Qin-Feng Zhu, Zhen Wang, Chun-Li Zhang, Xin Yang, Jin-Juan Zhang, Fu-Rui Wang, Jun Liu, Meng Zhou, Yong-Lin Wang, Xun He, Li-She Gan, Shang-Gao Liao

**Affiliations:** 1 State Key Laboratory of Functions and Applications of Medicinal Plants & School of Pharmacy, Guizhou Medical University, Guiyang 550025, China; xguobo@163.com (G.-B.X.); zhuqinfeng@gmc.edu.cn (Q.-F.Z.); chunlizhang0016@163.com (C.-L.Z.); yangxinxin20210721@163.com (X.Y.); zjj216@163.com (J.-J.Z.); FuruiWang66@163.com (F.-R.W.); Jun_Liu555@163.com (J.L.); 2 Engineering Research Center for the Development and Application of Ethnic Medicine and TCM, Ministry of Education & Guizhou Provincial Key Laboratory of Pharmaceutics, Guizhou Medical University, Guiyang 550004, China; zhoumeng@gmc.edu.cn (M.Z.); gywyl@gmc.edu.cn (Y.-L.W.); 3 College of Pharmacy, Jinan University, 601 West Huangpu Avenue, Guangzhou 510632, China; wangzhen0502@jnu.edu.cn; 4 College of Pharmaceutical Sciences, Zhejiang University, 866 Yuhangtang Rd., Hangzhou 310058, China

**Keywords:** *Pseudostellaria heterophylla*, pseudosterins, cardioprotective agent

## Abstract

*Pseudostellaria heterophylla* is used in China not only as a functional food but also as an herb to tonify the spleen, enhance immunity, and treat palpitation. Our previous investigation showed that a fraction enriched in glycosides obtained from the roots of *P. heterophylla* possessed pronounced protective effects on H9c2 cells against CoCl_2_-induced hypoxic injury. However, the active compounds responsible for the observed effects were still unknown. In the current investigation, pseudosterins A–C (**1**–**3**), three new alkaloids with a 1-ethyl-3-formyl-β-carboline skeleton, together with polydatin, have been isolated from the active fraction. Their structures were elucidated on the basis of spectroscopic analysis and quantum chemical calculations. The four compounds showed cardioprotective effects against sodium hydrosulfite-induced hypoxia-reoxygenation injury in H9c2 cells, with the three alkaloids being more potent. This is also the first report of alkaloids with a β-carboline skeleton isolated from *P. heterophylla* as cardioprotective agents.

## 1. Introduction

Cardiovascular diseases (CVDs) are a leading cause of death and disability worldwide, with an estimate of 17.9 million deaths every year [1]. As one of the most serious CVDs, myocardial infarction (MI), which is defined by pathology as myocardial cell death due to prolonged ischemia, was reported with over 700,000 deaths every year in China [2,3]. Effective treatment of MI (e.g., reperfusion therapy) generally involves procedures to promote the return of blood flow to the ischemic zone of the myocardium. However, reperfusion itself may aggravate myocardial damage and may lead to further irreversible myocardial cell death (i.e., lethal myocardial reperfusion injury) [4,5]. Protection of myocardium against ischemia/reperfusion (I/R) injury is, therefore, crucial in the process of reperfusion. Unfortunately, no effective therapy is currently available for combined I/R injury on the market, yet clinical trials in the past 10 years demonstrated that some chemical entities (e.g., cyclosporine A and metoprolol) were effective in ameliorating the myocardial damage [6,7,8]. Therefore, the development of powerful cardioprotective agents or functional foods to limit the extent of infarcted tissue caused by I/R injury is of great clinical importance.

**Figure 1:**
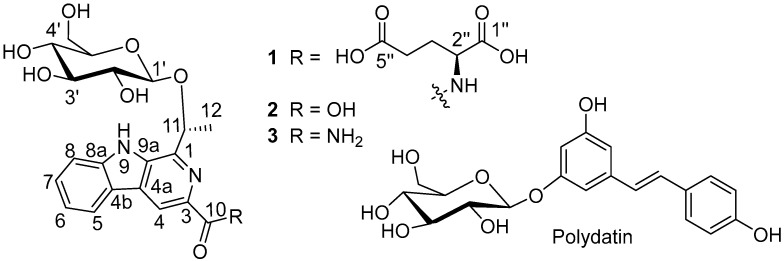
Structures of pseudosterins A–C (**1**–**3**) and polydatin.

The roots of *Pseudostellaria heterophylla* (Taizisheng in Chinese) were used not only as an herb to tonify the spleen, enhance immunity, and treat palpitation in Chinese herbal medicine (CHM) and local ethnic medicines [9,10,11,12], but also as a tonic food. A number of functional foods prepared from *P. heterophylla* (e.g., TaizishengHuangjing capsule^®^ and Taiziseng Tea (Hubei Zhenhao Biological Engineering Co., LTD, Yixing, China)) have been put into the market. Studies showed that *P. hetetophylla* is rich in a variety of chemical components, including polysaccharides, glycosides, cyclic peptides, sterols, oils, and other volatile oily substances with a wide range of bioactivities [13,14,15]. Previous pharmacological investigations showed that the extracts of *P. heterophylla* possessed cardioprotective effects [16,17,18,19]. Recently, we found that pretreatment of H9c2 cells with a fraction enriched in glycosides markedly protected the cells from CoCl_2_-induced hypoxic injury with effects comparable to that of the positive control *N*-Acetyl-l-Cysteine (NAC), and that the fraction may protect the cardiomyocytes from oxidative injury by preventing the increase of oxidative stress [20]. However, the active compounds responsible for the observed effects were still unknown. In the current investigation, three new alkaloids (**1**–**3**, Figure 1) with a 1-ethyl-3-formyl-β-carboline skeleton have been isolated from the active fraction. Their structures were elucidated on the basis of spectroscopic analysis and quantum chemical TDDFT calculations. Biological evaluations indicated that the three alkaloids showed more potent protective effects against sodium hydrosulfite-induced oxidative injury in H9c2 cells than that of polydatin, which was justified as an active principle in many fruits and vegetables [21,22,23]. These β-carboline alkaloids may be a promising type of compounds for the intervention of CVDs.

## 2. Results and Discussion

### 2.1. Structures Elucidation

**Figure 2:**
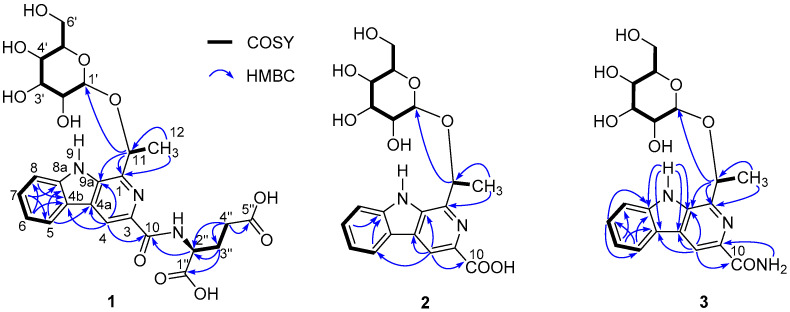
Key ^1^H-^1^H COSY and HMBC correlations of pseudosterins A–C (**1**–**3**).

Pseudosterin A (**1**) was obtained as a gum and possessed a molecular formula of C_25_H_29_N_3_O_11_ as determined by the HR-ESI-MS peak at *m*/*z* 548.1868 [M + H]^+^ (calcd 548.1875)(see Figure S3), corresponding to thirteen degrees of unsaturation. The IR absorptions suggested the presence of hydroxy (3086 cm^−1^), carboxylic (ca. 3000–2500 cm^−1^), carbonyl (1718 cm^−1^), and aromatic (1594 and 1498 cm^−1^) functionalities (see Figure S5 and Table S4). In the ^1^H-NMR spectrum data of **1** (CD_3_OD, see Table 1 and Figure S6), signals for the following substructures were observed: A 1,2-disubstituted benzene [δ_H_ 8.23 (1H, d, *J* = 7.8 Hz), 7.63 (1H, d, *J* = 8.2 Hz), 7.57 (1H, dd, *J* = 8.2, 7.4 Hz), and 7.30 (1H, dd, *J* = 7.8, 7.4 Hz)], and a CH_3_CH(O-)- fragment [δ_H_ 5.63 (1H, q, *J* = 6.6 Hz) and 1.81 (3H, d, *J* = 6.6 Hz)]. A combination of the remaining ^1^H-NMR signals with ^13^C-NMR and HSQC data (see Table 1 and Figures S7–S9) suggested the presence of a hexose [δ_H_ 4.52 (1H, d, *J* = 7.7 Hz), 3.45 (1H, dd, *J* = 8.3, 7.7 Hz), 3.36–3.33 (2H, m), 3.28–3.25 (1H, m), 3.93 (1H, dd, *J* = 11.8, 2.2 Hz), 3.74 (1H, dd, *J* = 11.8, 5.8 Hz); δ_C_ 102.9, 78.1, 78.1, 75.7, 71.7, 62.7]. However, one downfield singlet at δ_H_ 8.74 (1H, s) as well as five upfield signals [δ_H_ 4.71 (1H, br s), 2.48 (2H, m), 2.38 (1H, m), 2.18 (1H, m)] remained unassigned. In addition to signals for the above-deduced subunits that contained a hexose and a 1,2-disubstituted benzene [δ_C_ 122.6 (C-4b), 122.5 (C-5), 121.4 (C-6), 129.8 (C-7), 113.3 (C-8), 142.8 (C-8a)], the ^13^C-NMR and DEPT spectra showed additional signals for eight sp^2^ carbons [three acyl carbons at δ_C_ 176.8 (C-5″), 175.8 (C-1″), and 167.7 (C-10), one methine at δ_C_ 114.4 (C-4), and four quaternary carbons at δ_C_ 136.0 (C-9a), 131.4 (C-4a), 145.6(C-1), 139.2(C-3)] and three sp^3^ carbons [one methine at δ_C_ 53.9 (C-2″), and two methylenes at δ_C_ 31.5 (C-4″) and 28.8 (C-3″)]. When comparing the ^13^C-NMR data with those of 1-methyl-β-carboline-3-yl-(piperidin-1-yl)methanone [24], ethyl 2-methyl-9*H*-pyrido[2,3-b]indole-3-carboxylate [25] and flazin [26], and taking into consideration the elemental constitution and carbon resonances, a β-carboline scaffold was supposed to be present in **1** through convergence of the 1,2-disubstituted benzene group (see Figure S4), two of the three nitrogen atoms, and the five non-acyl sp^2^ carbons. Two-dimensional NMR experiments (HSQC, ^1^H-^1^H COSY, and HMBC) were further carried out to confirm the conclusion and to reveal the details of the structure. HSQC allowed the assignments of protons to their bonding carbons, while ^1^H-^1^H COSY enabled the confirmation of 1,2-disubstituted benzene, CH_3_CH(O-)- fragment, and hexose groups drawn in bold bonds (see Figure 2 and Figures S10–S12). Moreover, ^1^H-^1^H COSY correlations from H-3″ (δ_H_ 2.38 and 2.18) to H-2″ (δ_H_ 2.48) and H-4″ (δ_H_ 4.71) indicated the presence of a spin system corresponding to C2″-C3″-C4″, while a glutamic acid fragment was proposed for C-1″ (δ_C_ 175.8) through C-5″ (δ_C_ 176.8) by HMBC correlations H-2″/C-1″ and H-3″/C-5″ and on the basis of biosynthetic origin consideration. In addition, HMBC correlations between H-8/C-6 and C-4b, H-7/C-5 and C-8a, H-6/C-8 and C-4b, H-5/C-7, C-8a, and C-4a, and H-4/C-4a, C-4b, and C-9a confirmed the β-carboline structure, while correlations of H-11 with C-1 and C-9a suggested the ethoxyl group to be connected to C-1 via a carbon-carbon bond, and HMBC correlation between H-4/C-10 indicated that C-10 is located at C-3 of the β-carboline structure. A 1-ethyl-3-formyl-β-carboline skeleton was therefore established for **1**. HMBC correlation between H-2″/C-10 indicated the glutamic acid fragment to be located at C-10 via an amide bond, while that between H-11/C-1′ suggested the linkage of the hexosyl group at C-11. The planar structure of compound **1** was, therefore, established. The hexose obtained from HCl hydrolysate of **1** was confirmed by comparing its optical rotation data with that of an authentic d-glucose and by GC with their aldononitrile acetated derivatives (see Figures S13–S15). Coupling constant of the anomeric proton at δ_H_ 4.52 (1H, d, *J* = 7.7 Hz) suggested that the glucose had a β-configuration. Meanwhile, Marfey’s method was applied to identify the absolute configuration of glutamic acid in compound **1**. The 1-fluoro-2,4-dinitrophenyl-5-l-leucinamide (l-FDLA) derivatives of glutamic acid in the acid hydrolyzate of **1** and standards of l- and d-glutamic acids were subjected to LC-ESI MS analysis (see Figures S16 and S17). The UHPLC retention time and molecular weight of the glutamic acid derivative of **1** were the same as those obtained for the derivative of l-glutamic acid. Thus, the configuration of C-2″ was identified as *S*. The configuration of C-11 in **1** was, therefore, the only remaining task for its structural elucidation.

Pseudosterin B (**2**) was obtained as a gum and the HR-ESI-MS peak at *m*/*z* 417.1302 [M−H]^−^ (calcd 417.1303) (see Figure S18) suggested its molecular formula to be C_20_H_22_N_2_O_8_ with 11 degrees of unsaturation. Its UV and IR spectra (see Figures S19 and S20) bore a resemblance to those of **1**, indicating the presence of similar functionalities. Except signals for the glutamic acid moiety, the ^13^C-NMR data of **2** are very close to those of **1** (see Table 1 and Figures S22–S23), suggesting a possible β-carboline skeleton and a hexose moiety for **2**. However, the poor solubility of **2** in CD**_3_**OD gave poor peak resolution, which retarded the full assignments of the NMR data, whereas ^1^H-NMR data recorded in pyridine-*d_5_* are well-resolved and the structure was established by detailed 2D NMR (^1^H-^1^H COSY, HSQC, and HMBC) data analysis (see Figures S21, S24–S26). As described for **1**, a 1-ethyl-3-formyl-β-carboline skeleton can be easily identified by ^1^H-^1^H COSY, HSQC, and HMBC correlation analyses (see Figure 2). Coupling constants between the 1,3-diaxial protons (H-2′/H-4′ and H-1′/H-3′/H-5′) suggested the presence of a β-glucopyranosyl group, the configuration of which was also consistent with the coupling constant (δ_H_ 5.19, 1H, d, *J* = 7.3 Hz) of the anomeric proton. HMBC correlation between H-1′ and C-11 placed the β-glucopyranosyl group at C-11. Chemical shift (δ_C_ 168.8), molecular formula, and IR analysis suggested a carboxylic acid group for C-10. The structure of **2**, except the configuration of C-11, was, therefore, established.

Pseudosterin C (**3**) was obtained as white amorphous powder. Its molecular formula was determined to be C_20_H_23_N_3_O_7_ from the quasi-molecular ion peak at *m*/*z* 418.1609 [M + H]^+^ (calcd. 418.1614) in the HR-ESI-MS (see Figure S27), indicating 11 degrees of unsaturation. The IR absorptions suggested the presence of hydroxy or/and amino (3443–3210 cm^−1^), -NH_2_ (1649 and 1562 cm^−1^), and aromatic (1625 and 1508 cm^−1^) groups (see Figure S29). The ^1^H-NMR data (see Table 1 and Figure S30) of **3** was very close to those of **2** in DMSO-*d_6_* (due to poor coupling splitting in pyridine-*d_5_*, **3** was detected in DMSO-*d_6_*); these observations suggested that compound **3** also possessed a similar 11-β-glucopyranosyl-β-carboline structure, which was also supported by a UV, ^13^C NMR, ^1^H-^1^H COSY, HSQC, and HMBC correlation (see Figure 2 and Figures S28, S31–S35). HMBC correlation between -NH_2_ (δ_H_ 7.98, 1H, d, *J* = 2 Hz; δ_H_ 7.45, 1H, d, *J* = 2 Hz) and C-10 (δ_C_ 166.9), as well as the IR analysis (1643 and 1562 cm^−1^), indicated the presence of a CO-NH_2_ group at C-10. Therefore, the planar structure of **3** was established with only the configuration of C-11 to be determined.

**Figure 3:**
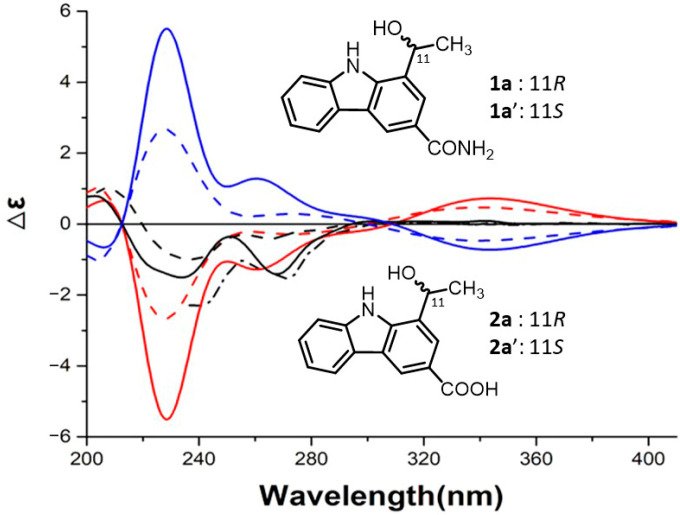
Experimental ECD spectra of **1** (black dash line), **2** (black solid line), **3** (black dash dot line), and B3LYP/6-311++G(2d,2p)//B3LYP/6-311++G(2d,2p) calculated ECD spectra (σ = 0.5, red shifted by 10 nm and σ = 0.4, red shifted by 15 nm) of **1a** (red dash lines) and **2a** (red solid lines), respectively, and calculated ECD spectra of enantiomers **1a**’ and **2a**’ (blue dash line and blue solid line, respectively).

The absolute configurations of C-11 in pseudosterins A–C (**1**–**3**) were determined by comparing their experimental ECD spectra with the quantum chemical TDDFT calculation-produced ECD spectra [27] of model compounds **1a** and **2a** (see Figure 3, Figure S1 and Table S1) that possess the chemical features of the chromophore and adjacent chiral carbons of the three compounds. In the experimental ECD spectra, three compounds showed a small positive first Cotton effect around 320 nm (see Figure 3, Tables S2 and S3), two negative ones around 270 and 235 nm, and ended by a positive one near 200 nm. Similarly, the calculated ones for the corresponding model compounds exhibited a positive first, negative second and third, and positive fourth Cotton effect. Since the glucose has already been determined to be d-glucose with GC analysis, NMR and optical rotation data all supported the assignment of compounds **1**–**3** to be a single compound but not a mixture of enantiomers, the theoretical and experimental ECD data qualitatively allowed the determination of the absolute configuration at C-11 of pseudosterins A–C (**1**–**3**) as *R*.

### 2.2. Hypoxia/Reoxygenation Injury Protective Activity

**Figure 4:**
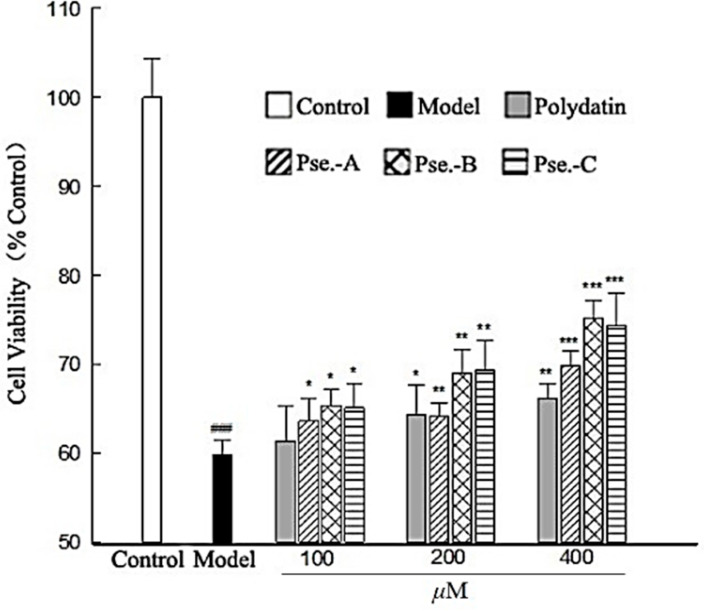
Cardioprotective effects of pseudosterins A–C and polydatin against sodium hydrosulfite-induced hypoxia-reoxygenation injury in H9c2 cells (*n* = 6). ### *p* < 0.001 vs. Control group; * *p* < 0.05, ** *p* < 0.01, *** *p* < 0.001 vs. Model group.

Since these compounds were obtained from the cardioprotective fraction, they were further evaluated for cardioprotective effects in H9c2 cells according to the method reported [28]. All the compounds showed no cytotoxicity at concentrations up to 500 μM (see Figure S2), whereas compounds **1**–**3** exhibited protective effects against Na_2_S_2_O_4_-induced hypoxia-reoxygenation injury in a concentration-dependent manner in the range of 100–400 μM (Figure 4), with a potency better than that of polydatin, a well-known natural cardioprotective agent that significantly decreased apoptosis rate in ischemia/reperfusion (I/R)-induced myocardial injury of rats [29,30] and that was also isolated as an active compound in the current investigation. The structure-activity relationship analysis showed that compounds **2** and **3**, which carry a free carboxylic or a primary amide group at C-3, are more active than compound **1**, which carries a secondary amide group. However, a negligible effect was observed when changing a free carboxylic group (as in **2**) to a primary amide one (as in **3**).

Myocardial ischemia/reperfusion (I/R) injury is a highly paradoxical injury in cardiovascular disease and its severity is proportional to the duration of ischemia. Reducing reperfusion injury would decrease the incidence of heart failure [31]. Myocardial cells exposed to hypoxia and reoxygenation (H/R) have been proved to be a useful tool for determining the biochemical responses of myocardial cells to I/R [32,33]. Sodium hydrosulfite is known as an oxygen scavenger, which was usually used as a chemical agent for establishing the myocardial cell model of hypoxia/reoxygenation [28,34]. In this study, pseudosterins A–C (**1**–**3**) effectively protected H9c2 cells from H/R injury induced by sodium hydrosulfite better than polydatin, which was proved to markedly alleviate the ischemic injury of cardiomyocytes from an animal model (30 mg/kg rat body weight, i.p.) [35]. Although extensive in vivo experiments of the three compounds are still needed for evaluating their cardioprotective effect, the current results suggested that they were possibly potent cardioprotective agents against myocardial I/R. Multiple mechanisms have been proved to be related to myocardial I/R injury. Reactive oxygen species (ROS) [36] and mitochondria [37,38] have been reported to be involved in myocardial I/R. Naturally occurring β-carbolines existing in foods (e.g., soy sources) and plants (e.g., *Peganum harmala*) are reported to be effective hydroxyl radical scavengers and antioxidants [39,40,41]. Therefore, the mechanisms of compounds **1**–**3** against Na_2_S_2_O_4_-induced H/R injury may be associated with ROS elimination. However, further research in this regard is needed.

## 3. Materials and Methods

### 3.1. General Experimental Procedures

High-resolution electrospray ionization mass spectra (HR-ESI-MS) were carried out on a Bruker Daltonics microTOF-Q II mass spectrometer (Bruker Daltoniks GmbH, Bremen, Germany) equipped with an ESI interface. UV spectra were obtained on a Perkin-Elmer S2 Lambda 35 UV/VIS spectrometer (PerkinElmer, Boston, MA, USA). IR spectra were detected by the Perkin-Elmer Spectrum One FT-IR spectrometer (as KBr pieces; in cm^−1^) (PerkinElmer, Boston, MA, USA). Optical rotations were measured on a Perkin-Elmer 341 polarimeter (PerkinElmer, Boston, MA, USA). NMR spectra were recorded on a Bruker AVANCE NEO 600 M (^1^H: 600 MHz; ^13^C: 150 MHz) (Bruker, Karlsruhe, Germany) or JEOL ECX 500/400 NMR spectrometer (^1^H: 500/400 MHz; ^13^C: 125/100 MHz) (JEOL, Akishima-shi, Japan). Thin-layer chromatography (TLC) was executed on silica gel GF_254_ from Qingdao Haiyang Chemical Co., Ltd, Qingdao, China (QHCC), detected under a UV lamp at 254 or 365 nm, and visualized by spraying with 10% sulfuric acid/ethanol (*v*/*v*) solution followed by heating. Column chromatography (CC) was performed on columns with silica gel (QHCC), Sephadex LH-20 (Pharmacia, Stockholm, Sweden), and semi-preparative high-performance liquid chromatography with a UV detector (Beijing Guopu Technology Co., LTD, Beijing, China) (λ = 254 nm) and a Kromasil C_18_ column (250 mm × 10 mm; 5 μm) (Akzo Nobel N.V, Stockholm, Sweden). All solvents were of analytical grade.

### 3.2. Plant Material

The roots of *Pseudostellaria heterophylla* (Miq.) Pax were collected in October 2011 from the Radix Pseudostellariae cultivation base, Shibing County, Guizhou province, China and were identified by Prof. Qing-De Long of Guizhou Medical University. A voucher specimen (accession No. 20111101) was deposited at the Herbarium of the School of Pharmacy, Guizhou Medical University.

Extraction and Isolation. The dried roots of *P. heterophylla* (30 kg) were powdered and extracted by boiling water (70 L, 1.5 h × 2 times). The extract was evaporated under reduced pressure to yield a dark brown residue and then re-dissolved in H_2_O. The obtained solution was then fractionated into five fractions, Fr. A–E, over a D101 macroporous resin (outer appearance: cream white opaque ball pellet resin; grain length: 0.3–1.25 mm) column eluted with 0, 30%, 50%, 70%, and 95% ethanol/water, respectively. According to the previously reported procedure [42], fraction B was re-dissolved/dispersed in water and successively extracted with ethyl acetate and *n*-butyl alcohol to give three fractions (ethyl acetate fraction, *n*-butyl alcohol fraction, and water fraction). Purification of the *n*-butyl alcohol fraction sequentially by silica gel (petroleum ether:acetone = 3:1→0:1) afford subfractions B1–B4. Fraction B2 was then subjected to Sephadex LH-20 eluted with MeOH to produce fractions B2a–B2c. Compound **1** (11 mg) was obtained from fraction B2b by Octadecylsilyl (ODS) column chromatography (CC) using MeOH–H_2_O (50:50, *v/v*) as a mobile phase. Subfraction B4 was also submitted to ODS CC eluted with MeOH-H_2_O (32:68, *v/v*) to yield compound **2** (5 mg). Fraction C was subjected to successive ethyl acetate and *n*-butyl alcohol extraction to give three fractions (Fr. C1–C3), Fr. C3 was sequentially separated by silica gel CC (CHCl_3_: MeOH = 10:1→1:1) to give Fr.C3a, Fr.C3b, and Fr.C3c. Purification of Fr.C3b by a Sephadex LH-20 CC (MeOH) yielded polydatin (12.1 mg), while reversed phase silica gel CC of Fr.C3c eluted with 30% MeOH in water gave compound **3** (5 mg).

### 3.3. Acid Hydrolysis, Derivatization, and GC Analysis

Pseudosterins A–C (**1**–**3**), 4.0, 3.0, and 3.0 mg, respectively) were hydrolyzed by 2 N HCl at 80 °C in wedge bottles. The sugar solutions were obtained after removal of impurity with dichloromethane, and the sugar was subsequently purified by the Sephadex LH-20 column. The obtained sugars were determined to be d-glucose by direct optical rotation comparison and co-TLC (BuOH/acetone/H_2_O, 4/3/1, Rf = 0.45) with that of an authentic d-glucose. Further derivatization and identification were conducted according to previous procedures [43,44]. Briefly, 1.2 mg hydroxylamine-HCl and 0.4 mg sugar samples (or standard d-glucose and d-mannose) were dissolved with 2 mL pyridine in batches in wedge bottles to react for 30 min at 90 °C. The solutions were then cooled to room temperature. After addition of 1 mL of acetic anhydride to react at 90 °C for 30 min, the mixture was cooled to room temperature. Chloroform (1 mL) was then added and the organic phase was washed twice with 1 mL of water. The obtained organic phase was dried with rotary evaporator, and then the residue was dissolved in 100 μL of ethyl acetate–hexane (1:1, *v/v*) for GC analysis. The GC analysis was performed on a 7890–5975C system equipped with flame ionization detector (FID) (Agilent Technologies Inc., Santa Clara, CA, USA) and HP-5 capillary column (30 m × 320 μm i.d., 0.25 μm film thickness). Samples (1 μL) were injected with a split ratio of 60:1 by the Agilent auto-injector. Helium was used as the carrier gas at a constant flow rate of 2 mL/min. The temperature program was set as follows: the initial column temperature of 180 °C was increased at 2 °C min^−1^ to 250 °C, and held for 2 min. The inlet temperature was 240 °C and the temperature of detector was 280 °C. All the sugar substructures of pseudosterins A–C (**1**–**3**) were finally identified as d-glucose via a comparison with an authentic substance.

### 3.4. Acid Hydrolysis, Derivatization, and LC-ESI MS Analysis

Compound **1** (1 mg) with 6 N HCl (200 μL) were heated at 115 °C for 16 h according to report to release glutamic acid [45]. This solution was evaporated to dryness, and the residue was dissolved in 50 μL of water. To the glutamic acid-containing solution was added 40 μL of 1 M NaHCO_3_ and then 120 μL of 1% L-FDLA in acetone. The solution was stirred and incubated at 40 °C for 2 h, then quenched by 40 μL of 1 N HCl. Finally, the solution was evaporated and re-dissolved with 500 μL of HPLC grade methanol for LC-ESI MS analysis. The corresponding l- and d-glutamic acids were modified in the same way. LC-ESI MS analysis was performed with an Ultimate 3000 UHPLC system (Thermo Fisher Scientific, Waltham, MA, USA) coupled with a TSQ endure mass spectrometer (Thermo Fisher Scientific, Waltham, MA, USA). The chromatographic column used was Agilent Eclipse Plus C18 (2.10 mm × 100 mm, 1.8 μm) maintained at 40 °C. Acetonitrile–0.1% formic acid aqueous solution was used as mobile phase under a linear gradient elution program (acetonitrile, 20%–40%) for 45 min with photodiode array detection. The flow rate was 0.2 mL/min and the injection volume was 5 μL. The following MS parameters were employed: the capillary voltage was set at 2.5 kV with the sheath and auxiliary nebulizing gas (N_2_) pressure set at 2 psi, respectively, and the vaporizer temperature was 275 °C. The scan range was set at *m*/*z* 50–1000. MS analysis was operated in the negative ion mode of electrospray ionization.

### 3.5. Hypoxia/Reoxygenation (H/R) Model and Experimental Protocols

The H/R model was established according to literature with minor modification [34]. Briefly, H9c2 cells were cultured in high glucose DMEM media supplemented with 10% (*v*/*v*) heat-inactivated fetal bovine serum (FBS), including penicillin (100 U/mL) and streptomycin (100 μg/mL). Cells grown at logarithmic growth stage were inoculated in a 96-well plate with a density of 4 × 10^4^ cells/mL (100 μL for each well) and cultured in an incubator with 5% CO_2_ at 37 °C for 24 h. The cells were then randomly divided into 14 groups: control group (with culture medium only), model group (with 20 mM of sodium hydrosulfite in the culture medium), and test groups (each tested compound had three groups with concentrations of 100, 200, and 400 μM of the compound in the culture medium, respectively). After removal of the supernatant, 100 μL of culture medium without (for blank and model groups) or with the tested compounds (for groups of polydatin and pseudosterins A–C, all purities above 95%) were added, and the mixtures were then cultured in an incubator for 24 h. Except for the blank group, the supernatant was removed and the cells were washed twice with sugar-free DMEM medium. The Na_2_S_2_O_4_ solution (20 mM) prepared with sugar-free DMEM medium was then added to the cells and incubated for 15 min to induce hypoxic condition in vitro. After removal of the Na_2_S_2_O_4_ solution from cells, high glucose DMEM medium was added and the cells were cultured for another 15 min of reoxygenation to mimic reperfusion. Cell viability was then determined by the MTS method. The OD values were measured with microplate reader at a wavelength of 490 nm. The cell survival rate was calculated as OD_test_/OD_control_ × 100%.

### 3.6. Statistical Analysis

Experimental data were shown as mean ± SD. The experiments were repeated three times. The difference between the mean values of two groups was assessed by the Student’s t-test. Multiple group comparisons were performed using a Dunnett’s test. The accepted level of significance for the test was *p* < 0.05. All statistical tests were carried out using the SPSS 19.0 for Windows.

## 4. Conclusions

In the current investigation, three β-carbolines with a 1-ethyl-3-formyl-β-carboline skeleton have been isolated as cardioprotective agents from *P. heterophylla* in the treatment of cardiovascular diseases and in its use as a tonic food. β-Carbolines are an important subclass of carboline alkaloids with prominent biological effects. Diverse bioactivities, including antimicrobial, antitumor, antiparasitic, anticonvulsant, and vasorelaxant activities, have intrigued chemists and pharmacologists over centuries since the isolation of harmalin in 1841, and nine β-carboline drugs have been commercialized [46,47,48,49]. Thus, the discovery of 1-ethyl-3-formyl-β-carbolines as a new type of cardioprotective agent would not only stimulate efforts toward their structure–activity relationship, pharmacological application, and/or chemical synthesis, but also promote the use of *P. heterophylla* as a functional food for patients with cardiovascular diseases.

## Figures and Tables

**Table 1:** ^1^H and ^13^C NMR data of pseudosterins A (**1**), B (**2**), and C (**3**).

No.	δ_H_ (*J* in Hz)			δ_C_		
	1 ^a^	2 ^b^	3 ^c^	1 ^a^	2 ^b^	3 ^c^
1				145.6	145.9	143.9
3				139.2	135.4	139.0
4	8.74 (1H, s)	9.34 (1H, s)	8.75 (1H, s)	114.4	117.0	113.2
4a				131.4	130.6	129.4
4b				122.6	124.1	120.9
5	8.23 (1H, d, 7.8)	8.31 (1H, br d, 7.4)	8.34 (1H, br d, 8.0)	122.5	122.4	121.9
6	7.30 (1H, dd, 7.8, 7.4)	7.35 (1H, br dd, 7.4, 7.3)	7.27 (1H, br dd, 8.0, 8.0)	121.4	120.6	120.0
7	7.57 (1H, dd, 8.2, 7.4)	7.55 (1H, br dd, 7.7, 7.3)	7.57 (1H, br dd, 8.0, 8.0)	129.8	128.9	128.5
8	7.63 (1H, d, 8.2)	7.77 (1H, br d, 7.7)	7.66 (1H, br d, 8.0)	113.3	112.9	112.4
8a				142.8	142.4	141.0
9		12.5 (1H, s)	11.76 (1H, s)			
9a				136.0	136.1	134.1
10				167.7	168.8	166.9
11	5.63 (1H, q, 6.6)	6.03 (1H, q-like)	5.48 (1H, q, 6.5)	78.4	77.4	76.9
12	1.81 (3H, d, 6.6)	1.97 (3H, d, 5.5)	1.74 (3H, d, 6.5)	21.7	22.3	21.4
1′	4.52 (1H, d, 7.7)	5.19 (1H, d, 7.3)	4.47 (1H, d, 7.7)	102.9	102.9	101.9
2′	3.45 (1H, dd, 8.3, 7.7)	4.29 (1H, dd, 7.9, 7.3)	3.25 (1H) ^o^	75.7	75.7	74.0
3′	3.34 (1H, m)	4.21 (1H, dd, 8.6, 7.9)	3.16 (1H) ^o^	78.1	78.5	77.1
4′	3.35 (1H, m)	4.33 (1H, br d, 8.6)	3.14 (1H) ^o^	71.7	71.7	70.2
5′	3.27 (1H, m)	3.92 (1H, br s)	3.18 (1H) ^o^	78.1	78.8	76.5
6′	3.93 (1H, dd, 11.8, 2.2) 3.74 (1H, dd, 11.8, 5.8)	4.53 (1H, br d, 11.4) 4.41 (1H, dd, 11.4, 4.6)	3.73 (1H, dd, 11.2, 5.1) 3.50 (1H, m)	62.7	62.6	61.1
1″				175.8		
2″	4.71 (1H, br s)			53.9		
3″	2.38 (1H, m) 2.18 (1H, m)			28.8		
4″	2.48 (2H, m)			31.5		
5″				176.8		
-CONH **^a^**			7.98 (1H, d, 2.0)			
-CONH **^b^**			7.45 (1H, d, 2.0)			

^a^ Recorded in CD_3_OD; ^b^ Recorded in C_5_D_5_N; ^c^ Recorded in DMSO-*d_6_*; ^o^ overlapped.

## Data Availability

The data presented in this study is available in Supplementary Material.

## Supplementary Materials

Supplementary Materials are available online. Tables S1–S3, and Figure S1: ECD calculation details; Table S4: Optical rotation, UV and IR data of compounds **1**–**3**; Figure S2: Cytotoxicity of compounds **1**–**3** and polydatin; Figures S3–S12: HR-MS, UV, IR, and 1D and 2D NMR of compound **1**; Figures S13–S15: Glucose fragment of **1**–**3** identification; Figures S16 and S17: Glutamic acid fragment of **1** identification; Figures S18–S26: HR-MS, UV, IR, and 1D and 2D NMR of compounds **2**; Figures S27–S35: HR-MS, UV, IR, and 1D and 2D NMR of compounds **3**. References [50,51,52] are cited in the Supplementary Material.

## Sample Availability

Samples of the compounds **1**–**3** are available from the authors.

## References

[1] Cardiovascular Diseases. http://www.who.int/en/news-room/fact-sheets/detail/cardiovascular-diseases-(cvds).

[2] Thygesen K., Alpert J.S., White H.D. (2007). Universal definition of myocardial infarction. J. Am. Coll. Cardiol..

[3] He Y., Li C., Ma Q., Chen S. (2018). Esculetin inhibits oxidative stress and apoptosis in H9c2 cardiomyocytes following hypoxia/reoxygenation injury. Biochem. Bioph. Res. Commun..

[4] Hausenloy D.J., Yellon D.M. (2013). Myocardial ischemia-reperfusion injury: A neglected therapeutic target. J. Clin. Investig..

[5] Zhao T.T., Yang T.L., Gong L., Wu P. (2018). Isorhamnetin protects against hypoxia/reoxygenation-induced injure by attenuating apoptosis and oxidative stress in H9c2 cardiomyocytes. Gene.

[6] Ibáñez B., Heusch G., Ovize M., Van de Werf F. (2015). Evolving therapies for myocardial ischemia/reperfusion injury. J. Am. Coll. Cardiol..

[7] Piot C., Croisille P., Staat P., Thibault H., Rioufol G., Mewton N., Elbelghiti R., Cung T.T., Bonnefoy E., Angoulvant D. (2008). Effect of cyclosporine on reperfusion injury in acute myocardial infarction. N. Engl. J. Med..

[8] Ibanez B., Cimmino G., Prat-González S., Vilahur G., Hutter R., Garcia M.J., Fuster V., Sanz J., Badimon L., Badimon J.J. (2011). The cardioprotection granted by metoprolol is restricted to its administration prior to coronary reperfusion. Int. J. Cardiol..

[9] Editorial Committee of Chinese Materia Medica, State Administration of Traditional Chinese Medicine (1999). Chinese Materia Medica (Zhonghua Bencao).

[10] Wu C., Lin Y. (2004). Advances in studies on Taizishen. J. Fujian Agri. Forest. Univ. (Nat. Sci. Edit.).

[11] Chen J. (2011). Edible development and prospects on *Pseudostellariae heterophylla*. Food Nutr. China.

[12] Zhang J., Yu H., You Y. (2011). The edible and medicinal value of Zherong radix pseudostellariae. Trait Pharm. J..

[13] You S., Liu X., Xu G., Ye M., Bai L., Lin R., Sha X., Liang L., Huang J., Zhou C. (2021). Identification of bioactive polysaccharide from *Pseudostellaria heterophylla* with its anti-inflammatory effects. J. Funct. Foods.

[14] Tan N.-H., Zhou J., Chen C.-X., Zhao S.-X. (1993). Cyclopeptide from the roots of *Pseudostellaria hetetophylla*. Phytochemistry.

[15] Fang Z.H., Duan X.C., Zhao J.D., Wu Y.J., Liu M.-M. (2018). Novel polysaccharide H-1-2 from *Pseudostellaria heterophylla* alleviates type 2 diabetes mellitus. Cell Physiol. Biochem..

[16] Shen X.C., Tao L., Peng J., Fang T.H. (2008). Effects of radix pseudostellariae on cardiac function and matrix metalloproteinases in rat chronic heart failure induced by coronary artery ligation. Chin. J. Pathophys..

[17] Xiao T.T., Peng J., Tao L., Shen X.C. (2012). Protection of effective fractions from pseudostellariae radix on primary cultured cardiac myocytes injury induced by norepinephreine in vitro. Chin. J. Exp. Tradit. Med. Formulae.

[18] Lin S.D., Dai Q.W., Zhang H.C., Hu J. (2010). Advances in chemical constituents and biological activities of radix pseudostellariae. Chin. J. Ethnomed. Ethnopharm..

[19] Shen X.C., Tao L., Bo S., Gan H.R., Duan J.A. (2008). Ameliorated effects of radix pseudostellariae on oxidative stress in rat chronic heart failure induced by acute cardiac infarction. West Chin. Med. J..

[20] Wang Z., Liao S.G., He Y., Li J., Zhong R.F., He X., Liu Y., Xiao T.T., Lan Y.Y., Long D.Q. (2013). Protective effects of fractions from *Pseudostellaria heterophylla* against cobalt chloride-induced hypoxic injury in H9c2 cell. J. Ethnopharmacol..

[21] Şöhretoğlu D., Baran M.Y., Arroo R., Kuruüzüm-Uz A. (2018). Recent advances in chemistry, therapeutic properties and sources of polydatin. Phytochem. Rev..

[22] Venturo N., Suffritti G. (2017). Improving dermatological signs and symptoms with polydatin-based dermocosmetic products. J. Cosmo. Trichol..

[23] Kammerer D., Claus A., Carle R., Schieber A. (2004). Polyphenol screening of pomace from red and white grape varieties (*Vitis vinifera* L.) by HPLC-DAD-MS/MS. J. Agric. Food Chem..

[24] Brahmbhatt K.G., Ahmed N., Sabde S., Mitra D., Singh I.P., Bhutani K.K. (2010). Synthesis and evaluation of β-carboline derivatives as inhibitors of human immunodeficiency virus. Bioorg. Med. Chem. Lett..

[25] Jiang Y., Yu S.-W., Yang Y., Liu Y.-L., Xu X.-Y., Zhang X.-M., Yuan W.-C. (2018). Facile synthesis of fused polycyclic compounds *via* intramolecular oxidative cyclization/aromatization of β-tetralone or β-tetralone oximes. Org. Biomol. Chem..

[26] Zhou H., Jian R., Kang J., Huang X., Li Y., Zhuang C., Yang F., Zhang L., Fan X., Wu T. (2010). Anti-inflammatory effects of caper (*Capparis spinosa* L.) fruit aqueous extract and the isolation of main phytochemicals. J. Agric. Food Chem..

[27] Zhou C.X., Zou L., Gan L.S., Cao Y.L. (2013). Kleinhospitines A-D, new cycloartane triterpenoid alkaloids from Kleinhovia hospital. Org. Lett..

[28] Ren C., Bao Y., Meng X.S., Diao Y.P., Kang T.G. (2013). Comparison of the protective effects of ferulic acid and its drug-containing plasma on primary cultured neonatal rat cardiomyocytes with hypoxia/reoxygenation injury. Pharmacogn. Mag..

[29] Du Q.H., Peng C., Zhang H. (2013). Polydatin: A review of pharmacology and pharmacokinetics. Pharm. Biol..

[30] Zhang L.P., Ma H.J., Bu H.M., Wang M.L., Li Q., Qi Z., Zhang Y. (2009). Polydatin attenuates ischemia/reperfusion-induced apoptosis in myocardium of the rat. Acta Physiol. Sin..

[31] Li X., Arslan F., Ren Y., Adav S.S., Poh K.K., Sorokin V., Lee C.N., Kleijn D., Lim S.K., Sze S.K. (2012). Metabolic adaptation to a disruption in oxygen supply during myocardial ischemia and reperfusion is underpinned bytemporal and quantitative changes in the cardiac proteome. J. Proteome Res..

[32] Kokura S., Yoshida N., Yoshikawa T. (2002). Anoxia/ reoxygenation-induced leukocyte-endothelial cell interactions. Free Radic. Biol. Med..

[33] Li C., Jackson R.M. (2002). Reactive species mechanisms of cellular hypoxia-reoxygenation injury. Am. J. Physiol. Cell. Physiol..

[34] Chu D., Zhang Z. (2018). Trichosanthis pericarpium aqueous extract protects H9c2 cardiomyocytes from hypoxia/reoxygenation injury by regulating PI3K/Akt/NO pathway. Molecules.

[35] Zhang L.P., Yang C.Y., Wang Y.P., Cui F., Zhang Y. (2008). Protective effect of polydatin against ischemia/reperfusion injury in rat heart. Acta Physiol. Sin..

[36] Granger D.N., Kvietys P.R. (2015). Reperfusion injury and reactive oxygen species: The evolution of a concept. Redox Biol..

[37] Chen Y.R., Zweier J.L. (2014). Cardiac mitochondria and reactive oxygen species generation. Circ. Res..

[38] Herraiz T., Galisteo J. (2015). Hydroxyl radical reactions and the radical scavenging activity of β-carboline alkaloids. Food Chem..

[39] Kim D.C., Quang T.H., Yoon C.S., Ngan N.T.T., Lim S.I., Lee S.Y., Kim Y.C., Oh H. (2016). Anti-neuroinflammatory activities of indole alkaloids from kanjang (Korean fermented soy source) in lipopolysaccharide-induced BV2 microglial cells. Food Chem..

[40] Wang K.B., Yuan C.M., Xue C.M., Li D.H., Jing Y.K., He H.P., Hao X.J., Di Y.T., Li Z.L., Hua H.M. (2014). Pegaharmalines A and B, two novel β-carboline alkaloids with unprecedented carbon skeletons from *Peganum harmala*. RSC Adv..

[41] Herraiz T., Guillén H. (2018). Monoamine oxidase—A inhibition and associated antioxidant activity in plant extracts with potential antidepressant actions. Biomed Res. Int..

[42] Zhang C.L., Xu G.B., Liu J., Liao S.G., He X. (2017). Chemical constituents of *Pseudostellaria heterophylla*. Nat. Prod. Res. Dev..

[43] McGinnis G.D. (1982). Preparation of aldononitrile acetates using N-methylimidazole as catalyst and solvent. Carbohyd. Res..

[44] Zhang W., He H., Zhang X. (2007). Determination of neutral sugars in soil by capillary gas chromatography after derivatization to aldononitrile acetates. Soil Biol. Biochem..

[45] Fujii K., Ikai Y., Oka H., Suzuki M., Harada K. (1997). A nonempirical method using LC/MS for determination of the absolute configuration of constituent amino acids in a peptide: Combination of Marfey’s method with mass spectrometry and its practical application. Anal. Chem..

[46] Goebel F. (1842). Über das harmalin. Eur. J. Org. Chem..

[47] Khan F.A., Maalik A., Iqbal Z., Malik I. (2013). Recent pharmacological developments in β-carboline alkaloid “harmaline”. Eur. J. Pharmacol..

[48] Khan H., Patel S., Kamal M.A. (2017). Pharmacological and toxicological profile of harmane-β-carboline alkaloid: Friend or Foe. Curr. Drug Metab..

[49] Dai J., Dan W., Schneider U., Wang J. (2018). β-Carboline alkaloid monomers and dimers: Occurrence, structural diversity, and biological activities. Eur. J. Med. Chem..

[50] Wavefunction Inc. (2018). Spartan 18.

[51] Frisch M.J., Trucks G.W., Schlegek H.B., Scuseria G.E., Robb M.A., Cheeseman J.R., Scalmani G., Barone V., Mennucci B., Petersson G.A. (2009). Gaussian 09.

[52] Stephens P.J., Harada N. (2010). ECD cotton effect approximated by Gaussian curve and other methods. Chirality.

