# Supplementary material for: Pseudosterins A–C, Three 1-Ethyl-3-formyl-β-carbolines from Pseudostellaria heterophylla and Their Cardioprotective Effects
Source: Molecules. 2021 Aug 20;26(16):5045. doi: 10.3390/molecules26165045 (PMC8398031; doi:10.3390/molecules26165045)
Supplement: Supplementary file 1 [file molecules-26-05045-s001.zip › molecules-1315741-supplementary.pdf]

molecules

MDPI

Supplementary Material

Pseudosterins A–C, Three 1-Ethyl-3-formyl-β-carbolines from *Pseudostellaria heterophylla* and Their Cardioprotective Effects

Guo-Bo Xu [1,2,†], Qin-Feng Zhu [1,†], Zhen Wang [3], Chun-Li Zhang [1], Xin Yang [1], Jin-Juan Zhang [1], Fu-Rui Wang [1], Jun Liu [1], Meng Zhou [2], Yong-Lin Wang [2], Xun He [1,*], Li-She Gan [4,*] and Shang-Gao Liao [1,2,*]

1 State Key Laboratory of Functions and Applications of Medicinal Plants and School of Pharmacy, Guizhou Medical University, Guiyang 550025, China; xguobo@163.com (G.-B.X.); zhuqinfeng@gmc.edu.cn (Q.-F.Z.); chunlizhang0016@163.com (C.-L.Z.); yangxinxin20210721@163.com (X.Y.); zjj216@163.com (J.-J.Z.); FuruiWang66@163.com (F.-R.W.); Jun_Liu555@163.com (J.L.)
2 Engineering Research Center for the Development and Application of Ethnic Medicine and TCM, Ministry of Education & Guizhou Provincial Key Laboratory of Pharmaceutics, Guizhou Medical University, Guiyang 550004, China; zhoumeng@gmc.edu.cn (M.Z.); gywyl@gmc.edu.cn (Y.-L.W.)
3 College of Pharmacy, Jinan University, 601 West Huangpu Avenue, Guangzhou, 510632, China; wangzhen0502@jnu.edu.cn
4 College of Pharmaceutical Sciences, Zhejiang University, 866 Yuhangtang Rd., Hangzhou 310058, China
* Correspondence: hexun224@gmc.edu.cn (X.H.); ganlishe@163.com (L.-S.G.); sgliao@gmc.edu.cn (S.-G.L.)
† These authors contributed equally to this work.

Content

S1. Experimental section ... 4
Figure S1. Lowest energy 3D conformers of model compounds ... 4
Table S1. Energy analysis ... 5
Table S2. ECD data ... 5
Table S3. ECD data ... 6
Table S4. Optical rotation, UV and IR data of pseudosterins A–C ... 7
Figure S2. Cytotoxicity of pseudosterins A–C (**1**–**3**) and polydatin ... 8
Figure S3. HR-MS of compound **1** ... 9
Figure S4. UV spectrum of compound **1** ... 9
Figure S5. IR spectrum of compound **1** ... 10
Figure S6. ^{1}H-NMR spectrum of compound **1** ... 10
Figure S7. ^{13}C-NMR spectrum of compound **1** ... 11
Figure S8. DEPT spectrum of compound **1** ... 11
Figure S9. HSQC spectrum of **1** ... 12
Figure S10. HMBC spectrum of **1** ... 12
Figure S11. HMBC spectrum of **1** ... 13
Figure S12. ^{1}H-^{1}H COSY spectrum of **1** ... 13
Figure S13. GC of D-glucose derivative ... 14
Figure S14. GC of D-mannose derivative ... 14
Figure S15. GC of sample ... 14
Figure S16. HPLC of glutamic acid derivatives ... 15
Figure S17. TIC of glutamic acid derivatives ... 15
Figure S18. HR-MS of compound **2** ... 16
Figure S19. UV spectrum of compound **2** ... 16
Figure S20. IR spectrum of compound **2** ... 17
Figure S21. ^{1}H-NMR spectrum of compound **2** ... 17
Figure S22. ^{13}C-NMR spectrum of compound **2** ... 18
Figure S23. DEPT spectrum of compound **2** ... 18
Figure S24. HSQC spectrum of **2** ... 19
Figure S25. HMBC spectrum of **2** ... 19
Figure S26. ^{1}H-^{1}H COSY spectrum of **2** ... 20
Figure S27. HR-MS of compound **3** ... 21
Figure S28. UV spectrum of compound **3** ... 21
Figure S29. IR spectrum of compound **3** ... 21
Figure S30. ^{1}H-NMR spectrum of compound **3** ... 22
Figure S31. ^{13}C-NMR spectrum of compound **3** ... 22

Figure S32. DEPT spectrum of compound **3** 23
Figure S33. HSQC spectrum of **3** 23
Figure S34. HMBC spectrum of **3** 24
Figure S35. ^{1}H-^{1}H COSY spectrum of **3** 24

S1. Experimental section

Procedures for the calculation of the ECD spectra of model compounds.

The absolute configuration at C-11 of compounds **1–3** were determined by their experimental ECD spectra and quantum chemical TDDFT calculations. Firstly, in order to avoid the large amount of lowest energy conformers brought by the flexible side chains and polyhydric glycosyl groups, which possessing known absolute stereochemistry and far from the chromophore, model compounds (**2a**, **1a,** see Figure S1) with the chemical features of the chromophore and adjacent chiral carbons were designed for each of the original compounds. Secondly, conformational analyses of these model compounds were carried out via Monte Carlo searching using molecular mechanism with MMFF force field in the *Spartan'18* program [50] The results showed three lowest energy conformers for each of **1a** and **2a**, (see Table S1), whose relative energy within 2 kcal/mol. Subsequently, the conformers were reoptimized using DFT at the B3LYP/6-311++G(2d,2p) level in vacuum with the Gaussian 09 program [51] The B3LYP/6-311++G(2d,2p) harmonic vibrational frequencies were further calculated to confirm their stability. The energies, oscillator strengths, and rotational strengths of the first 60 electronic excitations for **1a** and **2a** were calculated using the TDDFT methodology at the B3LYP/6-311++G(2d,2p) level in vacuum. The ECD spectra were then simulated by the overlapping Gaussian function [52] in which the velocity rotatory strengths of the first 32 electronic excitations for **1a**, the first 31 ones for **2a**. In order to get the final ECD spectrum of those compounds, the simulated spectra of the corresponding lowest energy conformers were averaged according to the Boltzmann distribution theory and their relative Gibbs free energy (ΔG).

Lowest energy Conformations

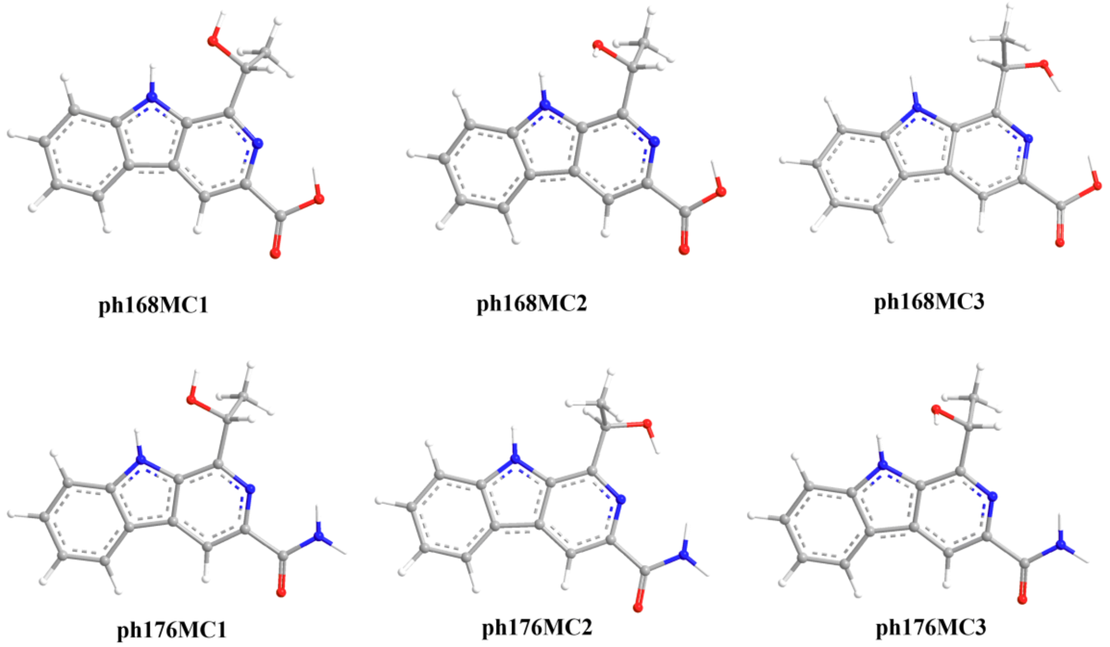

Figure S1. B3LYP/6-311++G(2d,2p) optimized lowest energy 3D conformers of model compounds.

Table S1. Energy analysis.

Conformation	MMFF energy		b3lyp/6-311++g(2d,2p) Gibbs Free Energy (298.15 K)		
	ΔE (kcal/mol)	Boltzmann Distribution	G (Hartree)	ΔG (kcal/mol)	Boltzmann Distribution
**1aC1**	0.00	0.819	−876.013499	0.08094879	0.456552977
**1aC2**	1.02	0.146	−876.013628	0	0.523428458
**1aC3**	1.88	0.034	−876.010548	1.9327308	0.020018565
**2aC1**	0.00	0.570	−856.129308	0.18448794	0.396342474
**2aC2**	0.51	0.240	−856.127564	1.27886538	0.062442038
**2aC3**	0.65	0.190	−856.129602	0	0.541215488

Table S2. ECD data.

State	1aC1		1aC2		1aC3	
	Excitation energies(eV)	Rotatory Strengths*	Excitation Energies(eV)	Rotatory Strengths*	Excitation energies(eV)	Rotatory Strengths*
1	3.8012	4.7607	3.8312	2.4813	3.8981	−5.3436
2	4.2388	−0.7786	4.2572	−1.0038	4.298	0.135
3	4.5776	−0.5588	4.568	−0.6138	4.5687	1.2928
4	4.7755	−4.0925	4.7424	1.5468	4.7731	14.6214
5	4.7815	1.7007	4.7797	0.6614	4.8315	15.4347
6	4.8592	−0.3869	4.8539	1.2283	4.8628	−12.7018
7	4.9273	0.1361	5.0364	0.8258	5.0839	−33.3147
8	5.1582	7.5547	5.1549	−26.1812	5.1053	0.0458
9	5.275	−1.4291	5.2485	18.7102	5.2545	19.3923
10	5.3607	−1.657	5.3214	−8.5259	5.3761	9.7463
11	5.3988	−12.435	5.4837	17.2547	5.4422	−3.362
12	5.5697	−3.6661	5.5117	−1.0432	5.5275	9.4325
13	5.5841	3.4253	5.5813	13.3856	5.6465	8.5838
14	5.6035	33.6904	5.6155	−3.4427	5.6807	48.4831
15	5.6502	−2.2707	5.666	−3.4754	5.7046	−10.7668
16	5.6742	−36.2401	5.7332	−14.8193	5.7323	−32.4065
17	5.8257	−8.6037	5.8371	−9.4754	5.8162	−0.4874
18	5.9315	−1.3797	5.9926	−3.4394	5.8706	−5.9361
19	5.9508	−8.1533	6.024	4.2938	6.0233	−0.9842
20	5.9867	−6.1988	6.0335	−0.4544	6.1267	16.5264
21	6.0128	3.1141	6.0512	6.6271	6.1453	−9.087
22	6.0253	4.6608	6.1819	3.5029	6.1871	−4.051
23	6.1381	0.6904	6.2087	5.6369	6.2609	−8.126
24	6.1868	4.1091	6.2371	9.5476	6.2674	7.8102
25	6.2201	−11.1423	6.2595	−6.7977	6.3399	−3.8841
26	6.2428	6.4013	6.2989	−2.3131	6.3528	−17.8545
27	6.3304	−2.0988	6.3299	−1.4597	6.3622	4.1105
28	6.3602	−0.0034	6.3401	0.1691	6.3837	3.2303
29	6.4148	−0.3046	6.4386	1.3156	6.4467	−3.8576
30	6.4231	−8.1527	6.4443	4.1052	6.4737	−0.8243
31	6.4407	1.6227	6.478	−12.1674	6.5149	28.2718
32	6.4994	18.8135	6.5028	−3.0172	6.556	−1.9602

33	6.5083	−21.1522	6.5461	−13.3181	6.6058	−0.1933
34	6.5567	27.8001	6.5844	−7.5957	6.6653	2.5152
35	6.5717	−10.9528	6.6523	−5.8623	6.6768	−6.5496
36	6.6195	0.9827	6.6696	9.6113	6.6974	18.7144
37	6.6248	−4.2713	6.6921	2.3816	6.7349	1.5101
38	6.6857	4.3641	6.7192	−6.1179	6.752	16.8689
39	6.7016	−8.7597	6.7557	1.4593	6.7812	−3.1205
40	6.725	−1.6094	6.7654	−19.3996	6.8256	1.1226
41	6.7428	3.2548	6.7839	4.969	6.8528	−19.1212
42	6.7602	−9.4994	6.8091	1.7411	6.866	−32.182
43	6.8327	8.7176	6.84	6.7232	6.8806	3.5903
44	6.8589	8.385	6.8607	3.1432	6.9116	0.5294
45	6.8764	4.7603	6.907	6.2048	6.9314	−6.1517
46	6.906	−9.1885	6.9574	−67.7593	6.9476	16.8023
47	6.917	−33.313	6.9723	7.9848	6.9801	−0.2709
48	6.9463	−3.9634	6.9841	22.6009	7.0117	−15.3456
49	6.9672	−2.5162	6.9914	8.3541	7.0167	5.8961
50	7.0034	11.5215	7.0089	34.8171	7.0752	−26.4168
51	7.0522	3.439	7.0329	34.1981	7.0966	69.7431
52	7.0666	−3.4505	7.0546	2.5961	7.1194	−17.9644
53	7.0861	1.6114	7.17	−7.8026	7.1602	−11.9597
54	7.1086	65.6653	7.1868	−0.1873	7.1995	3.6336
55	7.1199	−8.8504	7.1993	−15.8366	7.2522	4.2594
56	7.1582	1.2769	7.2298	−4.0257	7.2623	−4.4743
57	7.1643	4.7904	7.2454	−2.5895	7.2739	11.1518
58	7.184	−5.8515	7.2495	32.2959	7.2909	8.7992
59	7.2022	1.2631	7.2782	−2.2403	7.3105	−7.5397
60	7.2121	−18.4838	7.2974	2.1854	7.3207	−2.7956

* R(velocity) 10**−40 erg-esu-cm.

Table S3. ECD data (continued)

State	2aC1		2aC2		2aC3	
	Excitation energies(eV)	Rotatory Strengths*	Excitation energies(eV)	Rotatory Strengths*	Excitation energies(eV)	Rotatory Strengths*
1	3.8177	5.0497	3.9131	−6.256	3.8467	2.2346
2	4.3015	−0.9901	4.3604	2.0758	4.3174	−1.0539
3	4.4009	0.0841	4.4038	−1.654	4.3905	0.1758
4	4.5808	−0.0146	4.6061	−2.1693	4.5424	−0.1219
5	4.7742	−2.6528	4.821	23.5437	4.8051	2.5079
6	4.8188	−1.3647	4.9313	1.1354	4.9133	−0.1786
7	4.9326	−1.7337	4.9992	0.1381	4.9353	2.6969
8	5.0786	5.4006	5.0671	−14.3669	5.0532	−13.3467
9	5.1857	29.8426	5.1491	−32.3542	5.1016	−10.1775
10	5.191	−4.0275	5.2589	9.1634	5.1906	15.4882
11	5.2205	−17.8349	5.3532	45.4548	5.2992	−3.6746
12	5.2973	−12.0387	5.4239	−22.0494	5.3483	11.6306
13	5.4283	1.9812	5.5017	−1.1173	5.4447	−1.2187
14	5.4322	0.4747	5.5638	29.3874	5.51	13.8481
15	5.5414	0.5226	5.5948	−3.797	5.5354	17.0889
16	5.5892	−5.629	5.6159	−2.0573	5.5854	−20.3871

17	5.6152	−3.6364	5.6377	23.985	5.5986	−17.6057
18	5.6757	2.5196	5.6791	−11.2425	5.6769	−7.7997
19	5.7065	8.8204	5.714	−11.3841	5.7328	0.2278
20	5.7176	−4.4757	5.8845	−15.5119	5.8069	−2.3095
21	5.7514	−6.5571	5.9519	−7.3786	5.8469	5.9274
22	5.8186	−6.5407	5.9895	−0.4072	5.8943	3.8941
23	5.8415	−13.9375	6.0189	12.3482	5.944	1.7622
24	5.9801	−3.2466	6.0621	−3.9363	5.9946	2.3482
25	6.0168	0.927	6.0723	14.3842	6.0403	−6.0393
26	6.0368	−1.3767	6.1366	−8.7655	6.0966	2.7234
27	6.0727	−0.5938	6.1652	−6.3751	6.1389	7.419
28	6.102	1.5545	6.1779	−1.8061	6.1479	15.0132
29	6.1066	−0.9145	6.2037	−8.7289	6.1667	2.9334
30	6.1516	−6.3239	6.2387	1.0867	6.2132	−3.926
31	6.1884	3.6674	6.3307	10.3666	6.2537	−3.2054
32	6.2639	−2.5025	6.3433	9.5692	6.2678	−23.31
33	6.2866	−3.2674	6.4039	−8.7011	6.3384	1.3476
34	6.3138	0.2946	6.4311	14.5946	6.3632	−1.5322
35	6.3455	2.3429	6.4885	−3.2504	6.3916	−8.8568
36	6.356	1.8326	6.5032	−8.5134	6.4087	3.7164
37	6.3731	−3.255	6.5186	−1.052	6.4379	5.465
38	6.4295	14.5562	6.5364	37.8251	6.4601	2.9953
39	6.4556	−8.8014	6.5702	3.1207	6.4665	−13.891
40	6.4708	12.5967	6.5927	2.535	6.4909	21.6126
41	6.4953	−0.3212	6.6041	−5.0402	6.5527	−1.0428
42	6.5192	−18.3649	6.6545	−3.929	6.5692	3.3409
43	6.5242	9.3468	6.6658	6.8965	6.5816	−19.8525
44	6.5601	−0.3185	6.6924	−19.6487	6.5882	−16.5207
45	6.5967	7.8948	6.7092	10.6306	6.6141	5.2628
46	6.6104	−3.46	6.7176	1.8827	6.6327	0.2722
47	6.6366	−1.2294	6.7389	−26.3297	6.6413	−15.2639
48	6.6539	3.2584	6.7686	−2.6887	6.7044	−0.0888
49	6.6637	1.8649	6.7794	−11.4425	6.7172	−8.4394
50	6.6834	−26.7387	6.7955	−3.9418	6.7742	−4.125
51	6.7109	18.9257	6.8177	21.0063	6.7808	8.1434
52	6.7204	5.4011	6.8815	8.2286	6.7861	−0.3094
53	6.7789	−3.3959	6.9051	−18.0253	6.8164	1.8877
54	6.8136	3.5844	6.9133	−4.7967	6.8299	13.8364
55	6.8258	4.2454	6.926	−5.4641	6.8433	11.9945
56	6.8389	−19.1862	6.971	4.189	6.8516	0.9893
57	6.8674	6.1186	6.9984	−12.9208	6.89	54.9392
58	6.8814	8.8291	7.0036	−2.4952	6.9266	1.4142
59	6.887	−10.043	7.0361	37.1845	6.9507	0.7455
60	6.9399	−1.3866	7.0555	11.5527	6.9725	−0.1976

* R(velocity) 10**−40 erg-esu-cm.

Table S4. Optical rotation, UV and IR data of pseudosterins A–C.

Compound	Optical rotation	UV	IR (KBr)
Pseudosterin A	$[\alpha]_D^{29}$ −50 (*c* 0.33, H_2O)	UV (H_2O) λ_{max} (logε): 213 (3.44), 238 (3.52), 272 (3.69) nm	ν_{max}: 3086, 2935, 1870, 1846, 1718, 1653, 1624, 1594, 1538, 1498, 1467, 1351, 1249, 1123, 1075, and 1031 cm^{-1}.
Pseudosterin B	$[\alpha]_D^{29}$ −48 (*c* 0.42, H_2O)	UV (H_2O): 213 (3.40), 239 (3.37), 269 (3.55) nm	ν_{max}: 3124, 1717, 1595, 1541, 1522, 1560, 1457, 1374, 1259, 1138, 1122, 1067 cm^{-1}.

Pseudosterin C	$[\alpha]_D^{29}$ −11 (*c* 0.73, CH_3OH)	UV (CH_3OH): 219 (3.43), 239 (3.47), 272 (3.66) nm	ν_{max}: 3443, 3396, 3210, 2932, 2363, 1649, 1625, 1562, 1508, 1370, 1072, 1037, and 752 cm^{-1}.

Cytotoxicity of pseudosterins A-C and polydatin on H9c2 cells

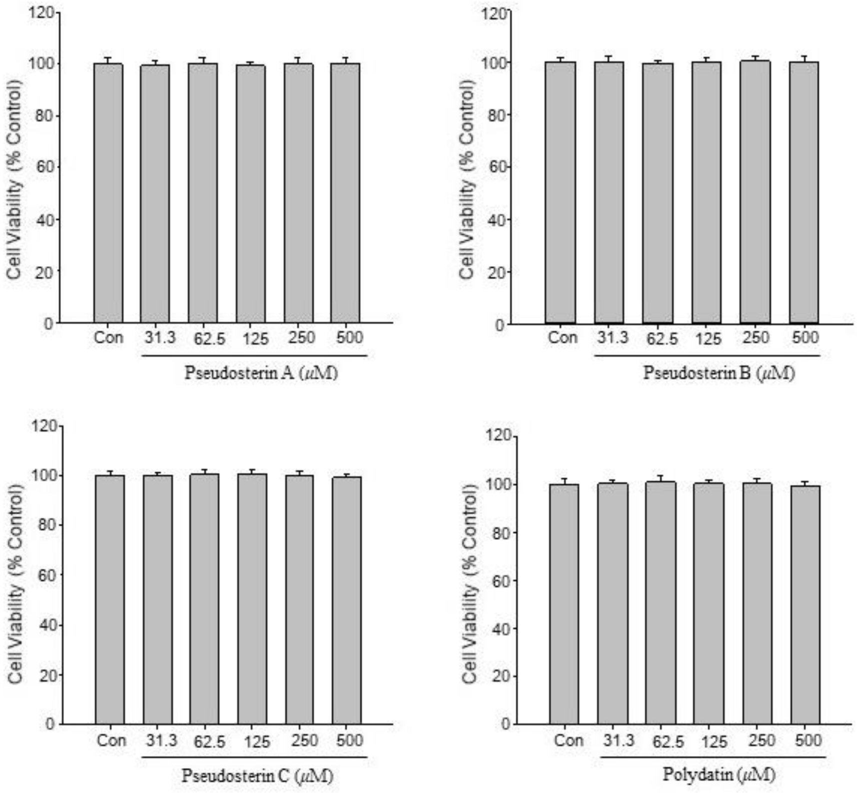

Figure S2. Cytotoxicity of pseudosterins A–C (**1**–**3**) and polydatin on H9c2 cells. $p > 0.05$ *vs* Control group ($n = 6$)

Acquisition Parameter

Source Type	ESI	Ion Polarity	Positive	Set Nebulizer	1.2 Bar
Focus	Not active	Set Capillary	3500 V	Set Dry Heater	200 °C
Scan Begin	100 m/z	Set End Plate Offset	-500 V	Set Dry Gas	8.0 l/min
Scan End	1500 m/z	Set Collision Cell RF	150.0 Vpp	Set Divert Valve	Waste

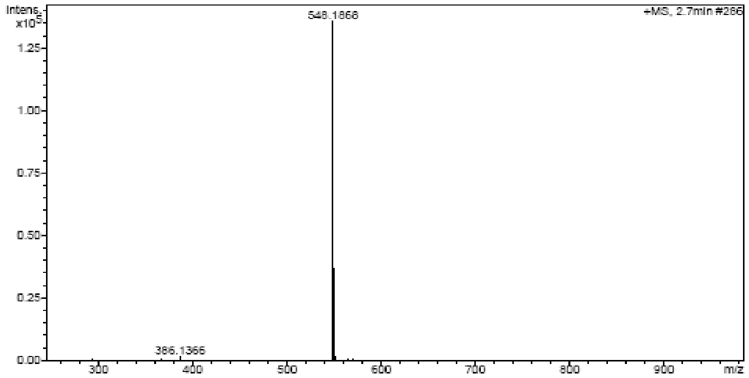

Meas. m/z	#	Formula	Score	m/z	err [ppm]	Mean err [ppm]	mSigma	rdb	e⁻ Conf	N-Rule
548.1868	1	C 25 H 30 N 3 O 11	100.00	548.1875	1.2	1.4	6.7	12.5	even	ok
	2	C 37 H 26 N O 4	15.63	548.1856	-2.1	-1.9	66.6	25.5	even	ok
	3	C 38 H 22 N 5	16.01	548.1870	0.3	0.5	79.4	30.5	even	ok

Figure S3. HE-MS of **1**

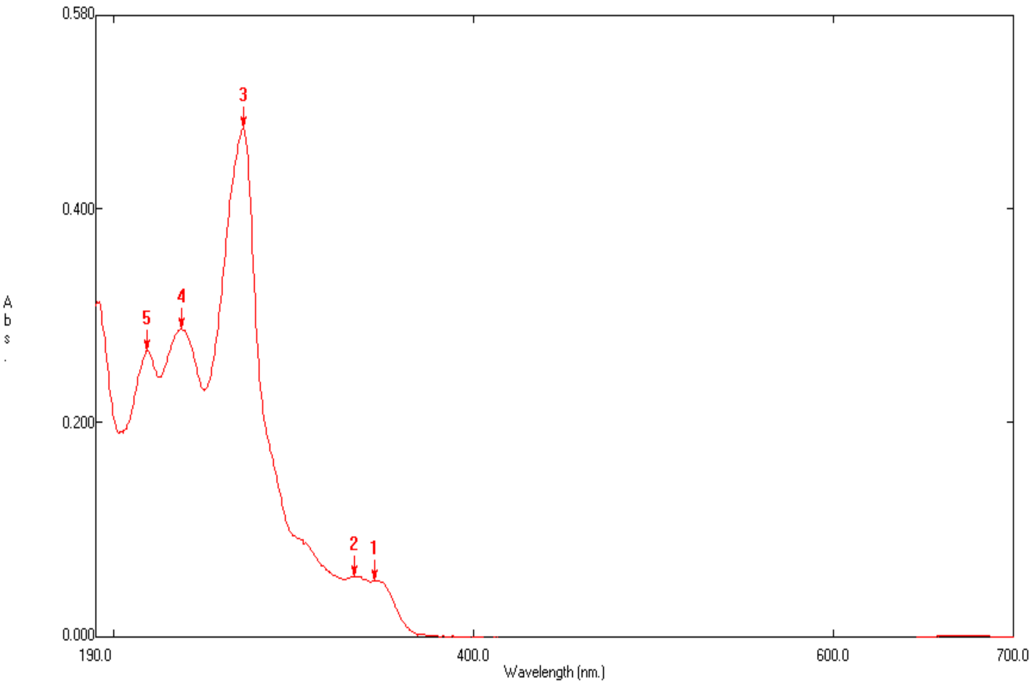

Figure S4. UV of compound **1**

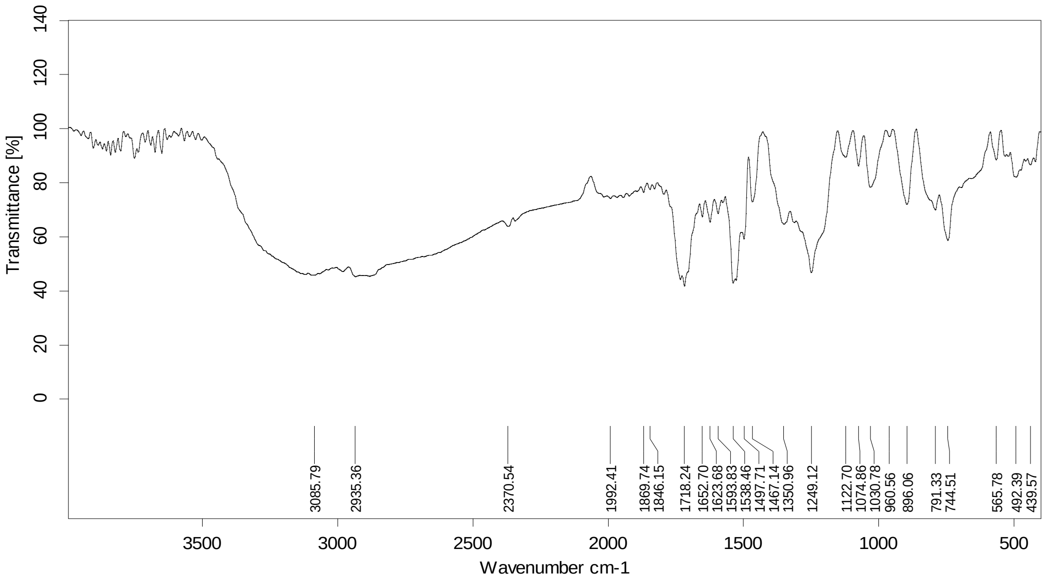

Figure S5. IR of compound **1**

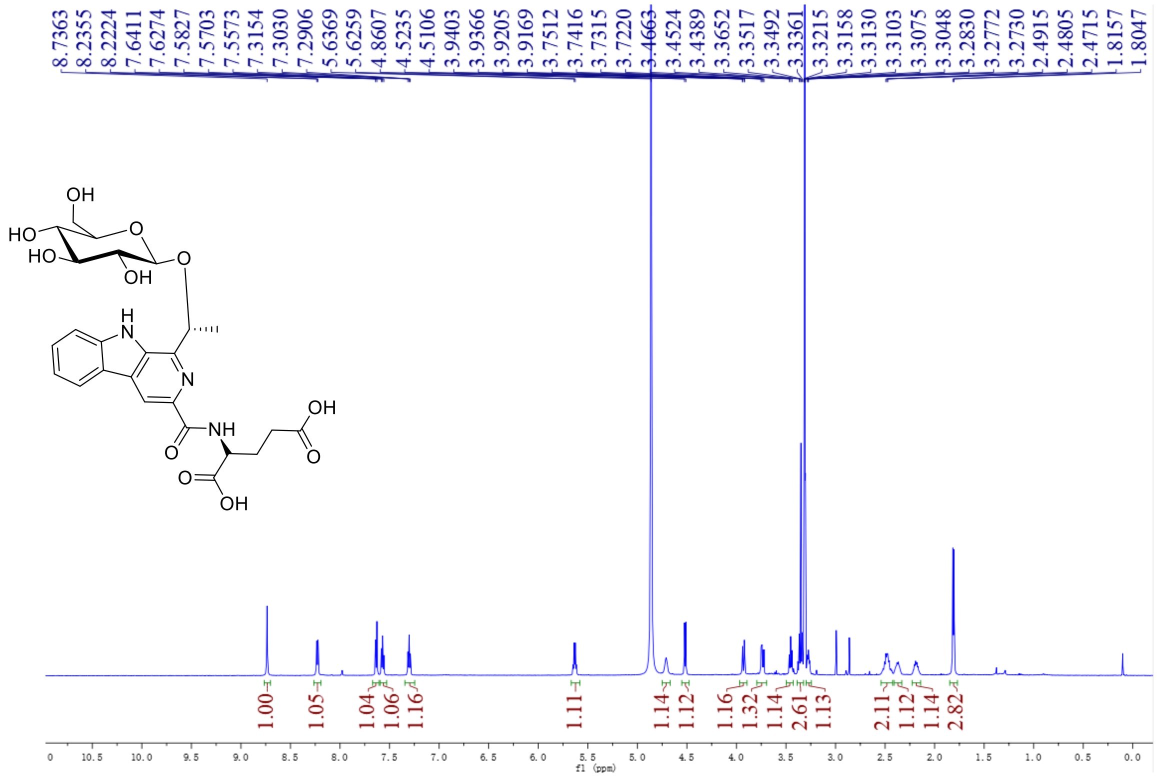

Figure S6. ^{1}H-NMR of compound **1** (CD_3OD, 600 MHz).

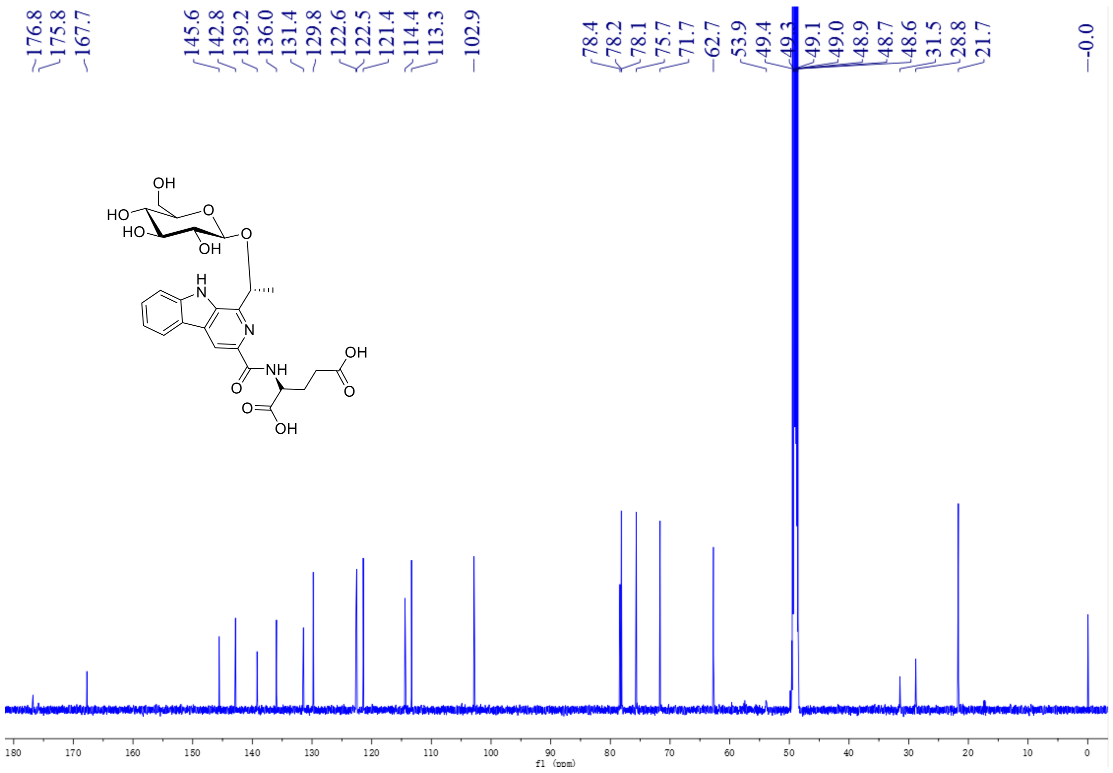

Figure S7. ^{13}C-NMR of compound **1** (CD_3OD, 150 MHz).

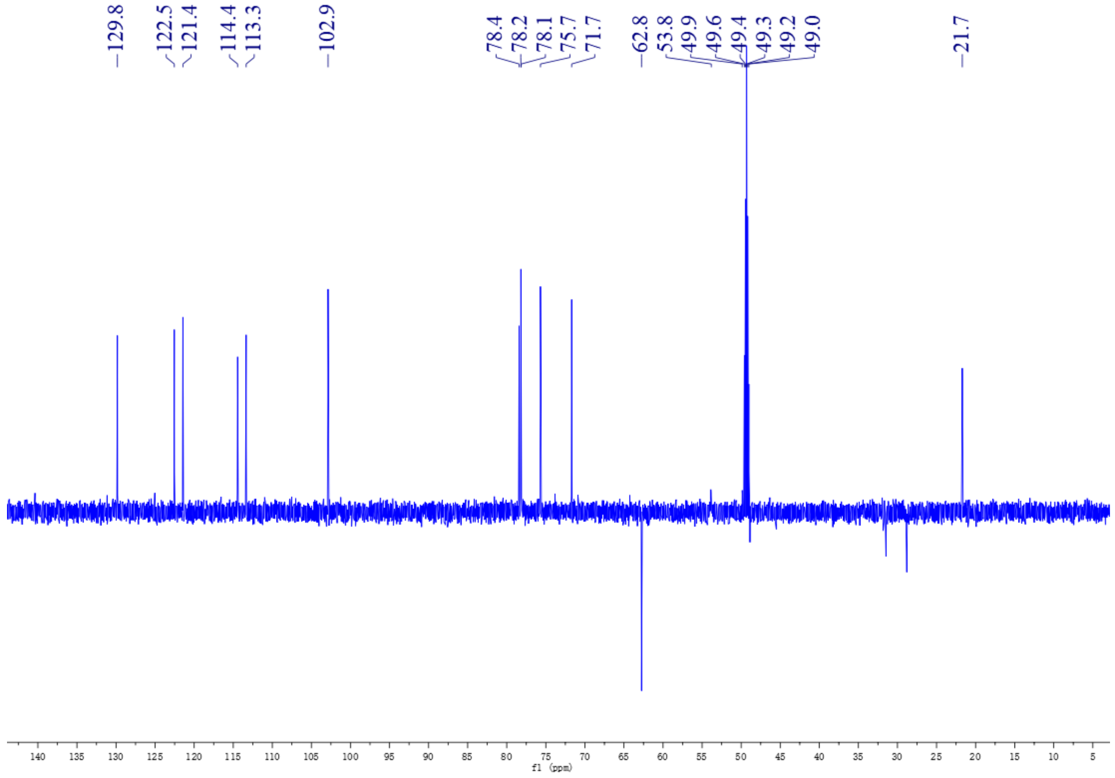

Figure S8. DEPT (135°) of compound **1** (CD_3OD, 150 MHz).

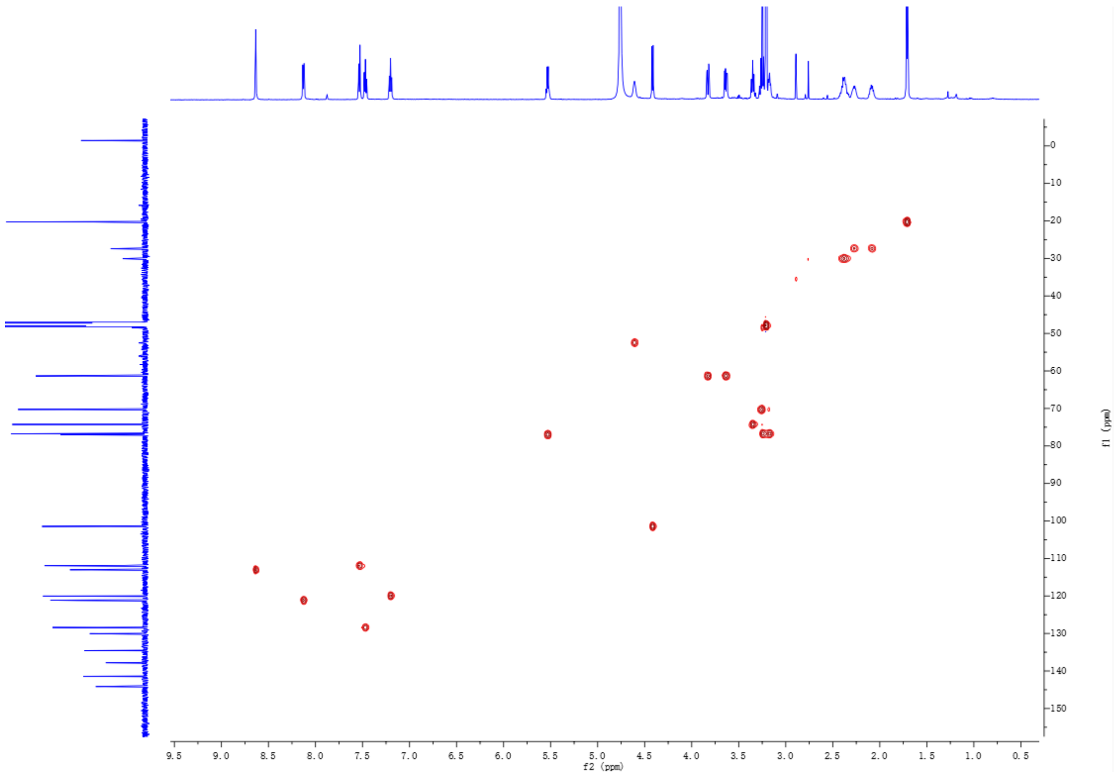

Figure S9. HSQC spectrum of **1** (CD_3OD, 600 MHz).

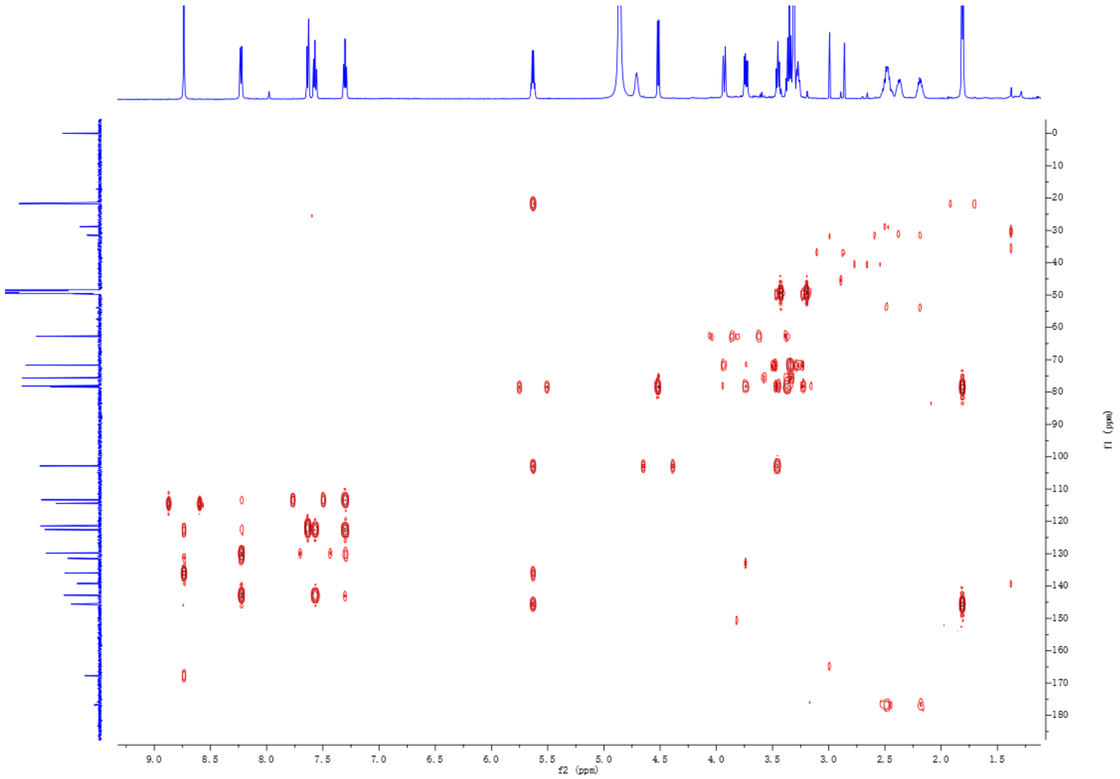

Figure S10. HMBC spectrum of **1** (CD_3OD, 600 MHz).

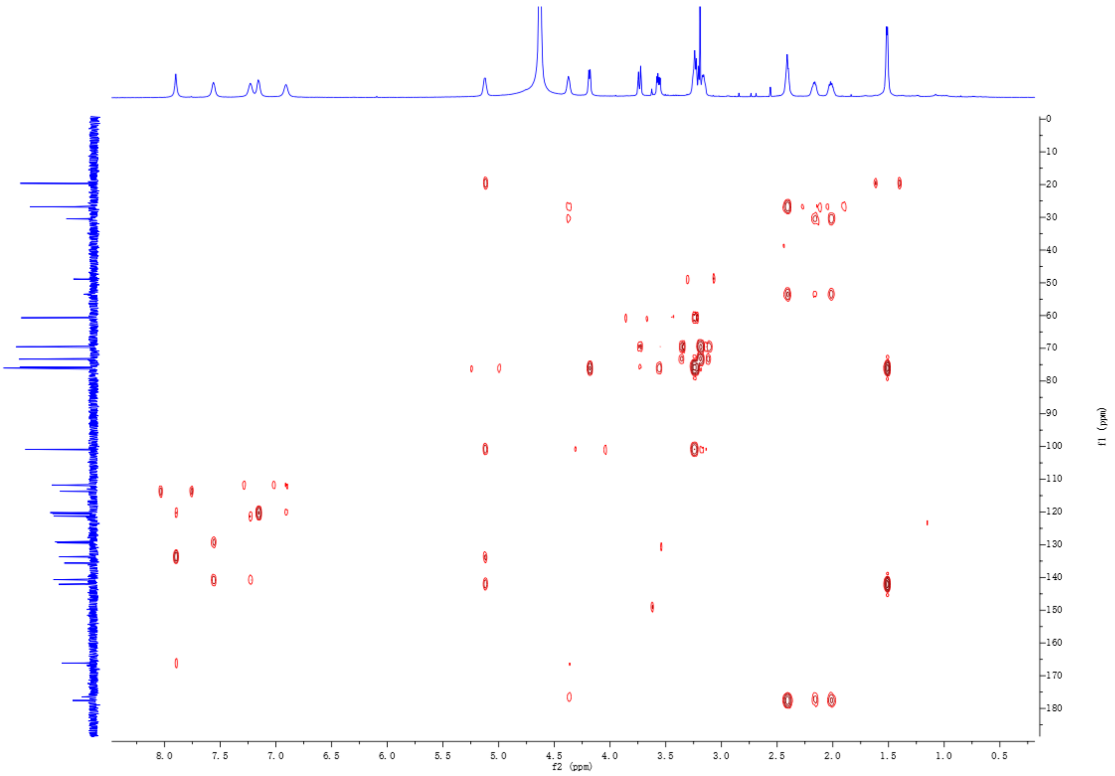

Figure S11. HMBC spectrum of **1** (D_2O, 600 MHz).

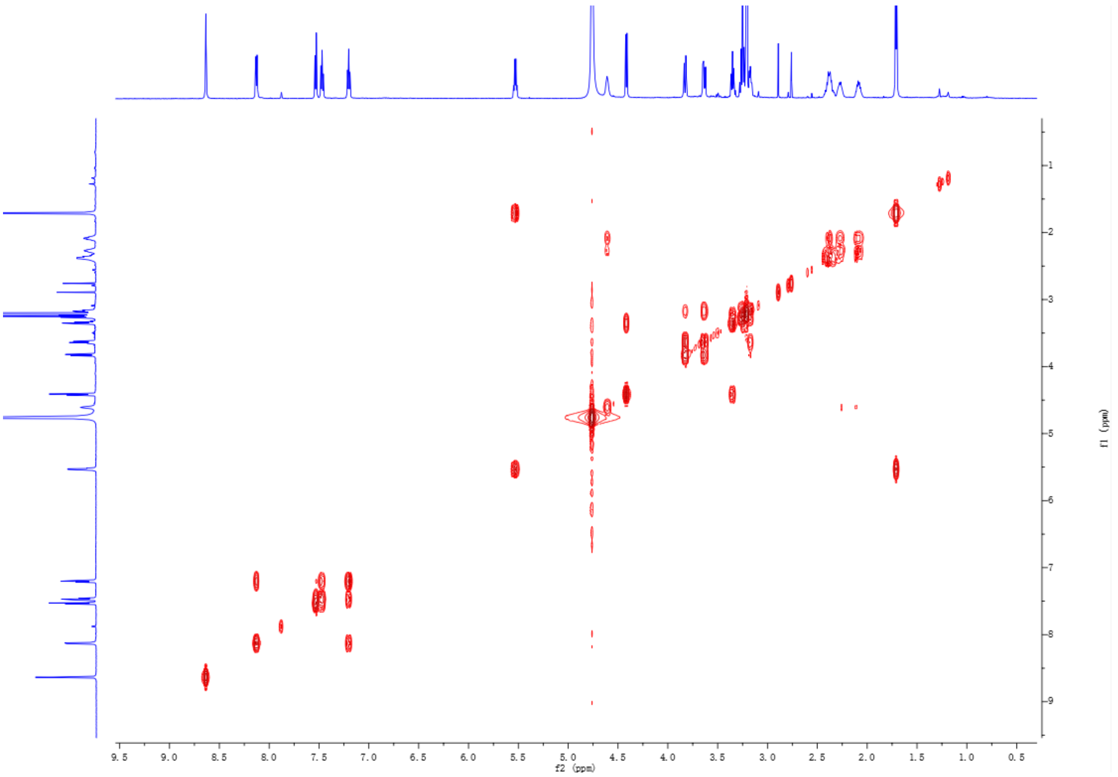

Figure S12. ^1H-^1H COSY spectrum of **1** (CD_3OD, 600 MHz).

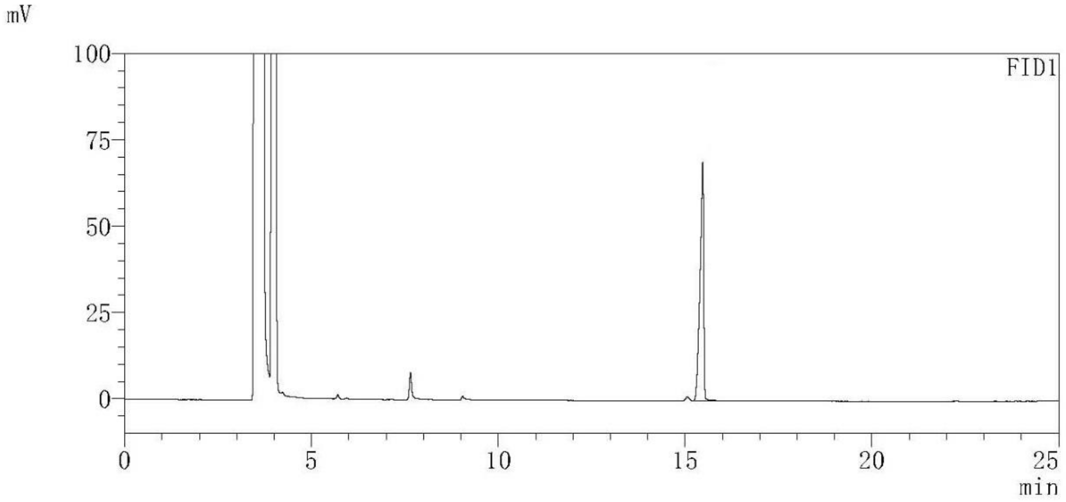

Figure S13. GC of D-Glucose derivative (Rt = 15.472 min).

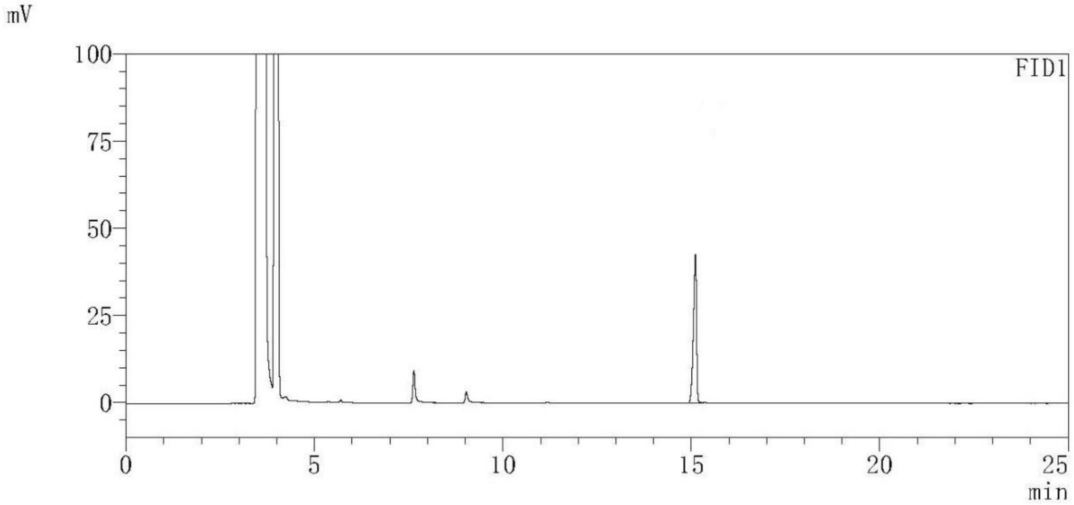

Figure S14. GC of D-mannose derivative (Rt = 15.111 min).

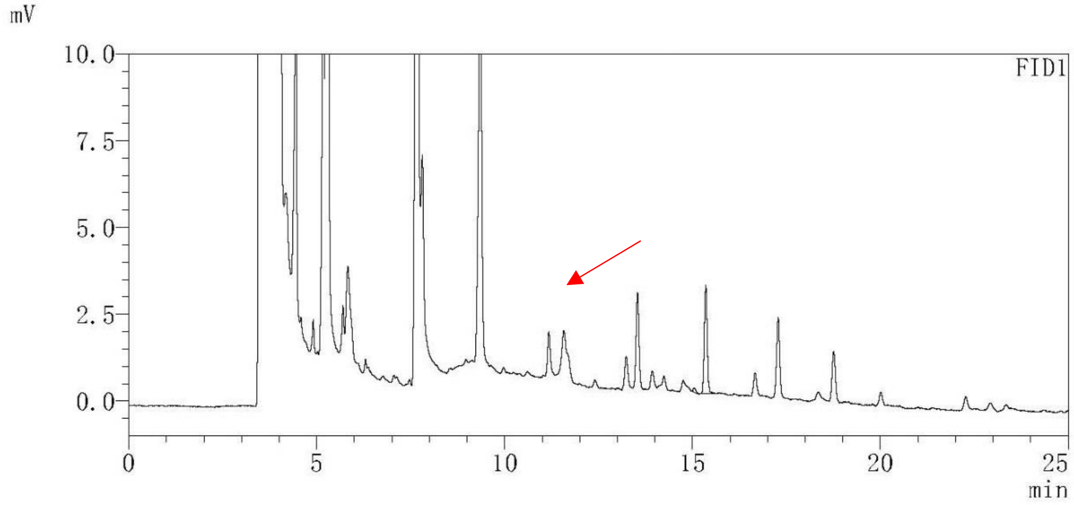

Figure S15. GC of sugar derivative from **1** after acid hydrolysis and derivatization (Rt = 15.460 min).

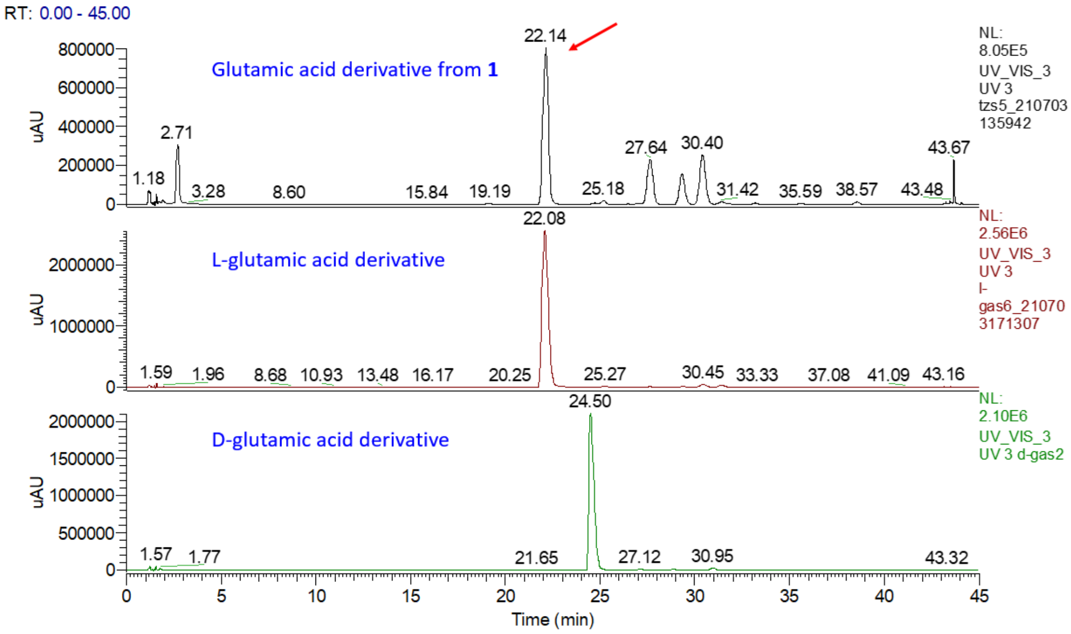

Figure S16. HPLC of glutamic acid derivatives.

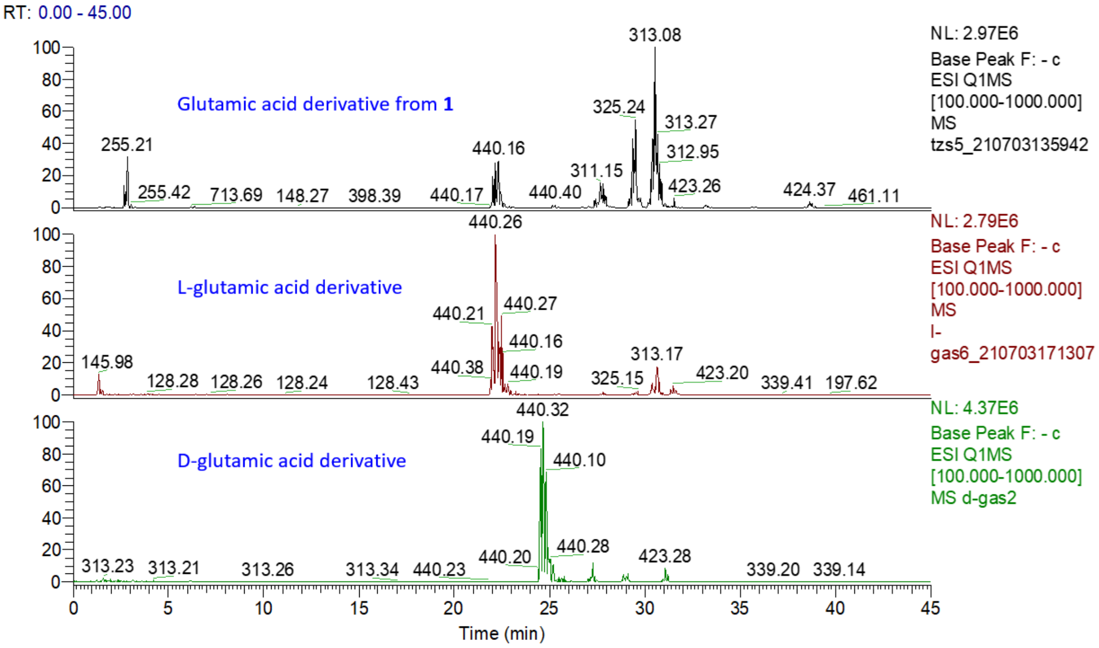

Figure S17. TIC of glutamic acid derivatives (negative ion mode, *m*/*z* 440 [M−H]$^-$).

Acquisition Parameter

Source Type	ESI	Ion Polarity	Negative	Set Nebulizer	1.2 Bar
Focus	Not active	Set Capillary	2800 V	Set Dry Heater	200 °C
Scan Begin	50 m/z	Set End Plate Offset	-500 V	Set Dry Gas	8.0 l/min
Scan End	1000 m/z	Set Collision Cell RF	150.0 Vpp	Set Divert Valve	Waste

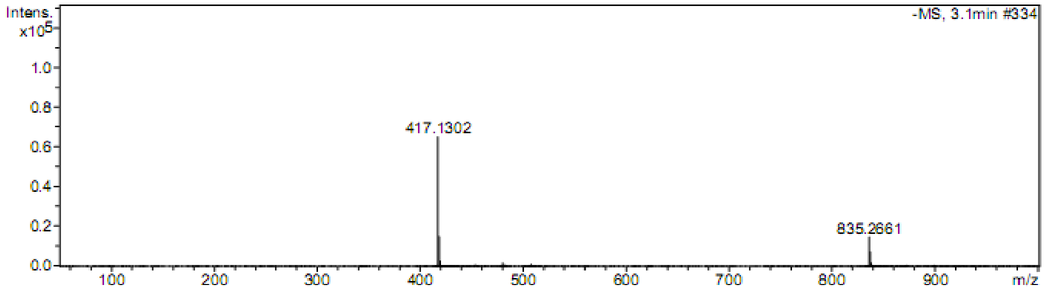

Meas. m/z	#	Formula	Score	m/z	err [ppm]	Mean err [ppm]	mSigma	rdb	e⁻ Conf	N-Rule
417.1302	1	C 20 H 21 N 2 O 8	100.00	417.1303	0.3	0.6	2.0	11.5	even	ok
	2	C 17 H 13 N 12 O 2	53.34	417.1290	-2.9	-2.8	6.7	17.5	even	ok
	3	C 21 H 17 N 6 O 4	40.44	417.1317	3.5	3.7	12.5	16.5	even	ok

Figure S18. HR-MS of compound **2.**

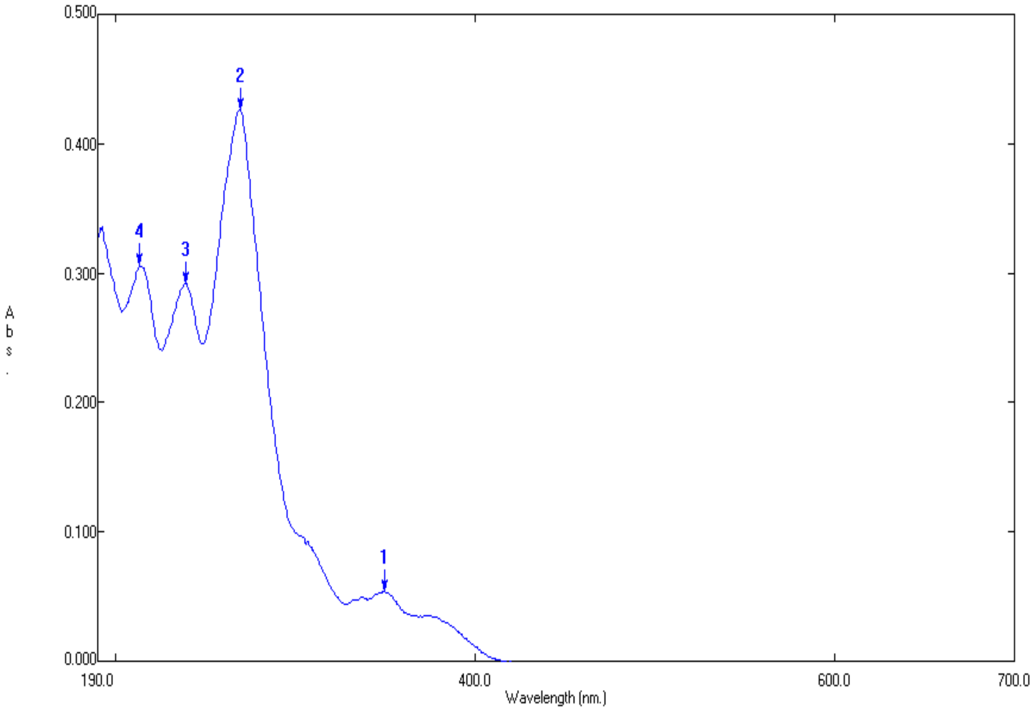

Figure S19. UV spectrum of compound **2.**

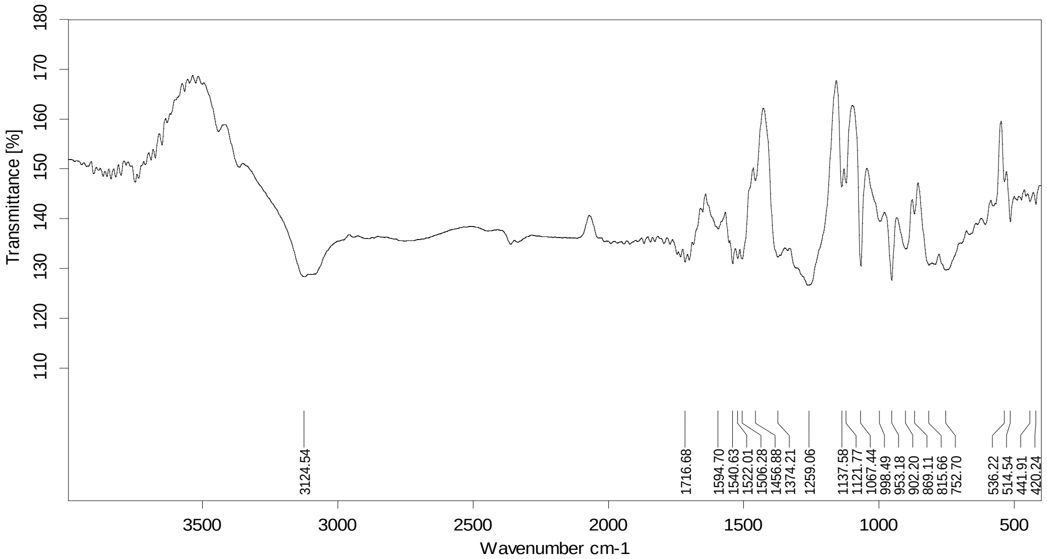

Figure S20. IR spectrum of compound **2.**

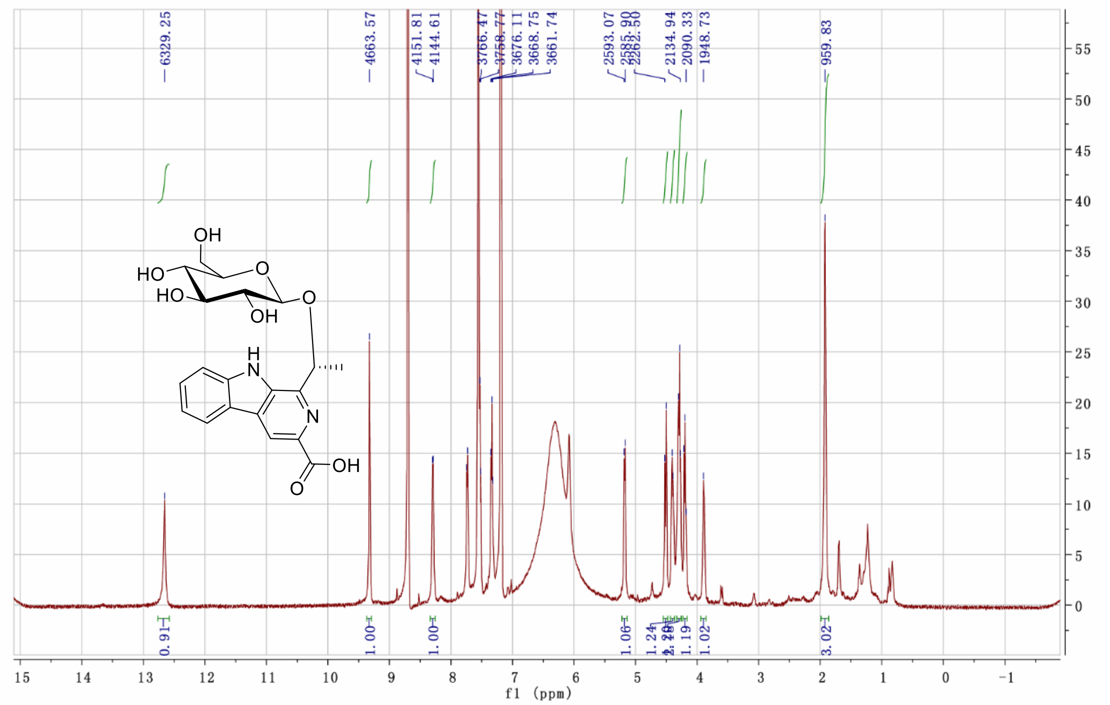

Figure S21. ^{1}H-NMR spectrum of compound **2** (pyridine-d_5, 500 MHz).

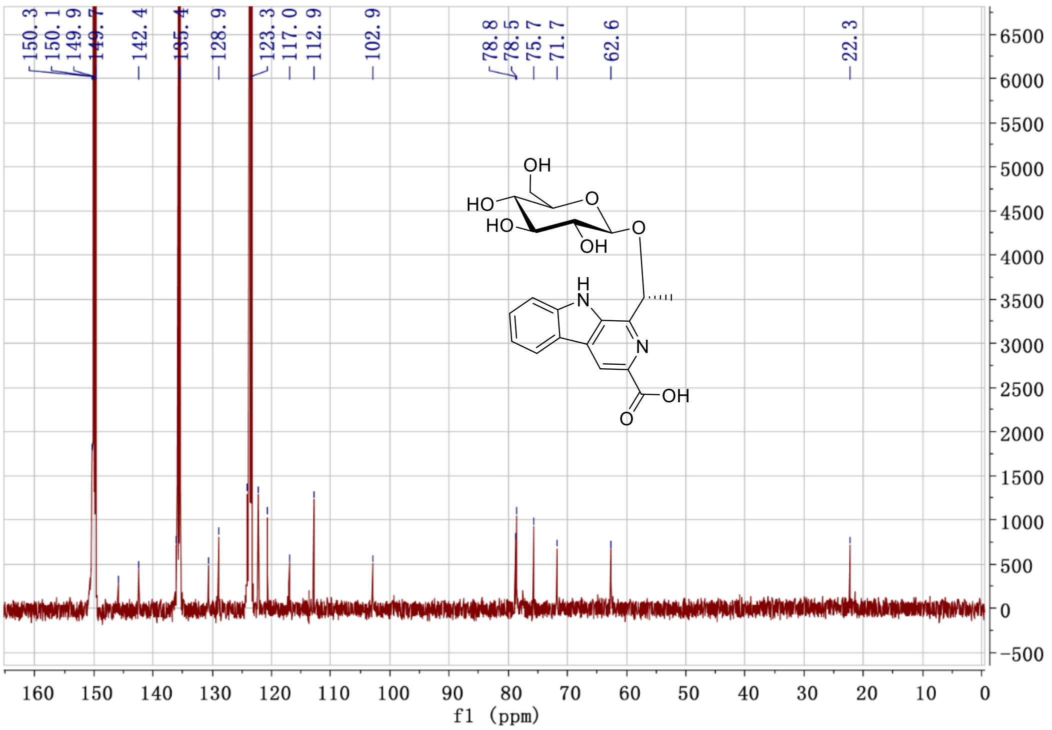

Figure S22. ^{13}C-NMR spectrum of compound **2** (pridine-*d*5, 125 MHz).

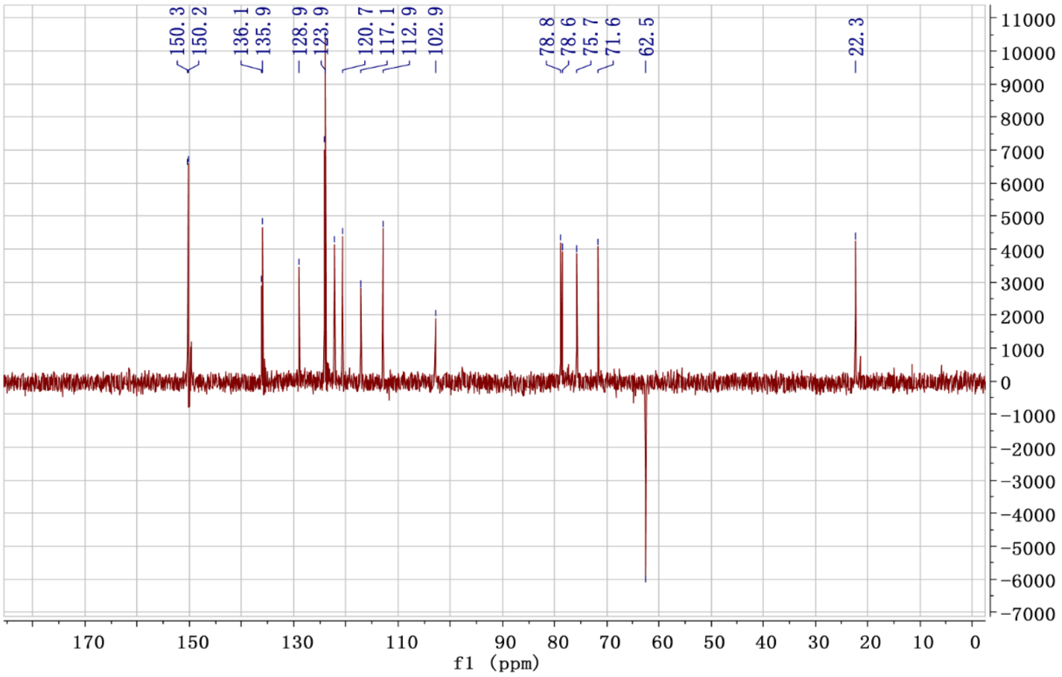

Figure S23. DEPT (135°) spectrum of compound **2** (pyridine-*d*5, 125 MHz).

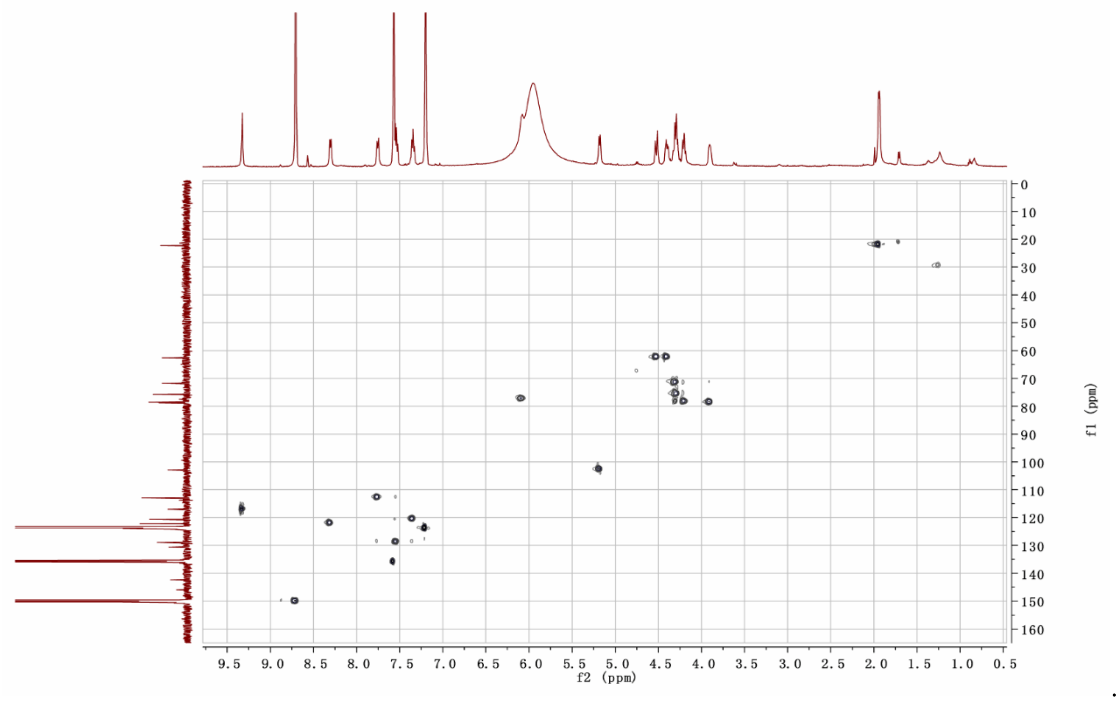

Figure S24. HSQC spectrum of **2** (pyridine-d_5, 500 MHz).

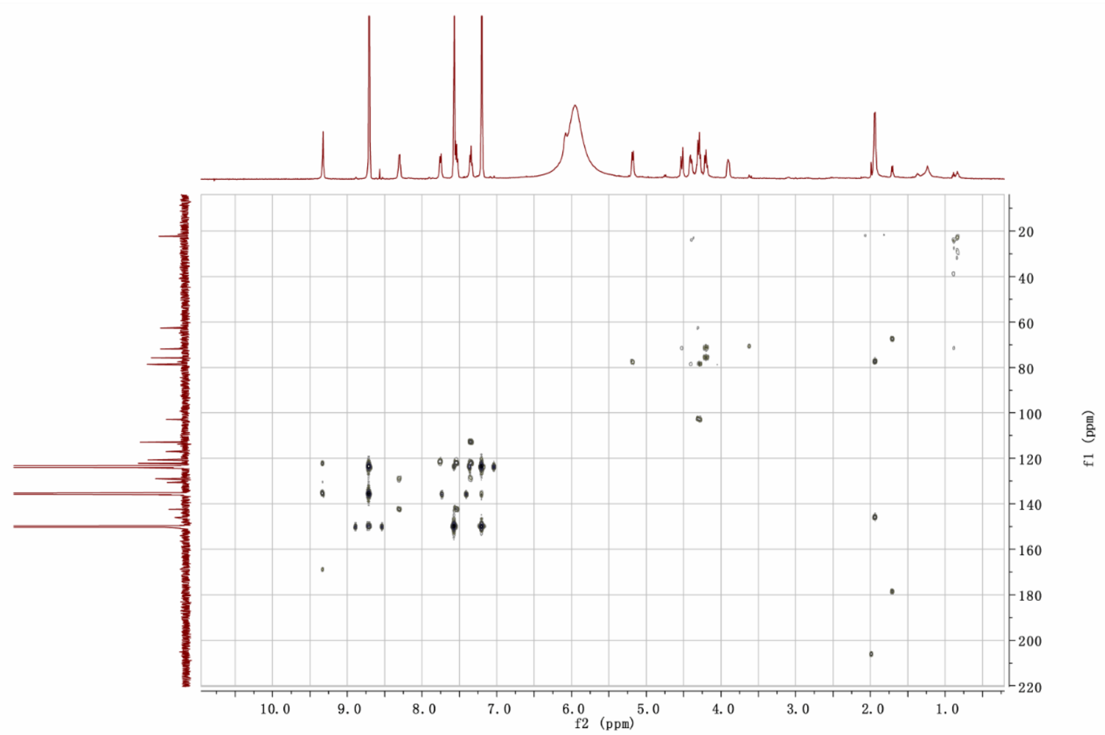

Figure S25. HMBC spectrum of **2** (pyridine-d_5, 500 MHz).

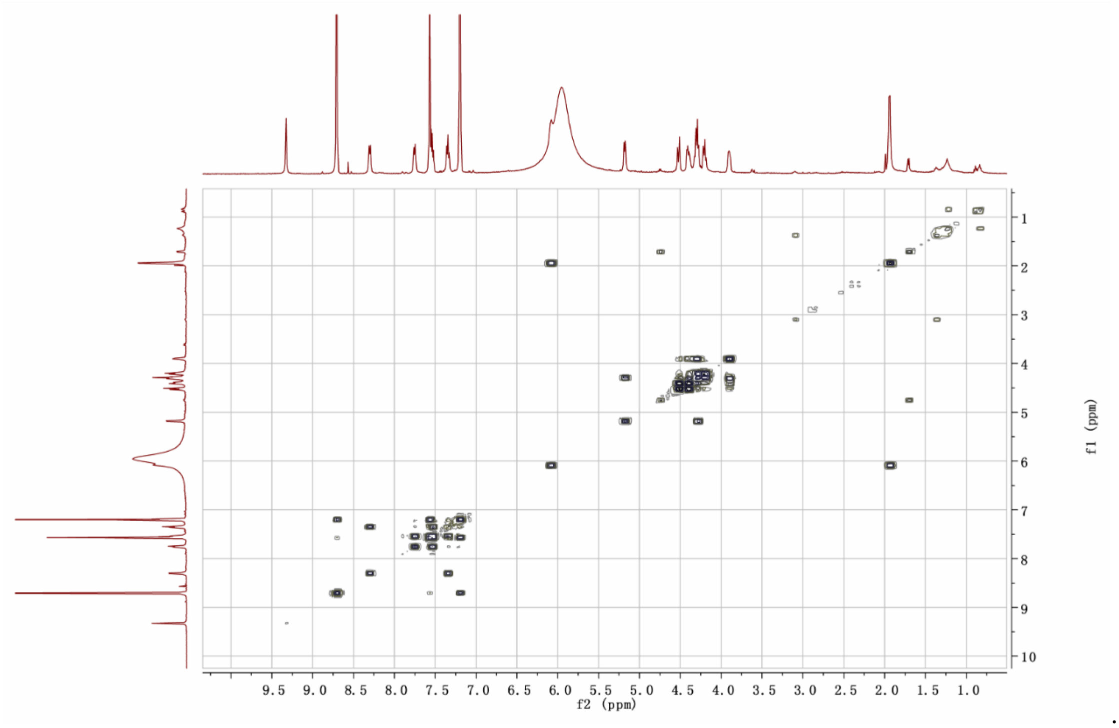

Figure S26. ^{1}H-^{1}H COSY spectrum of **2** (pyridine-*d*5, 500 MHz).

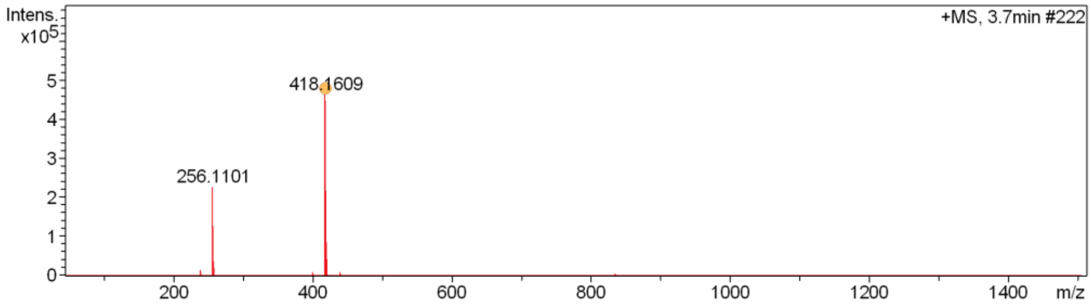

Figure S27. HR-MS of compound **3.**

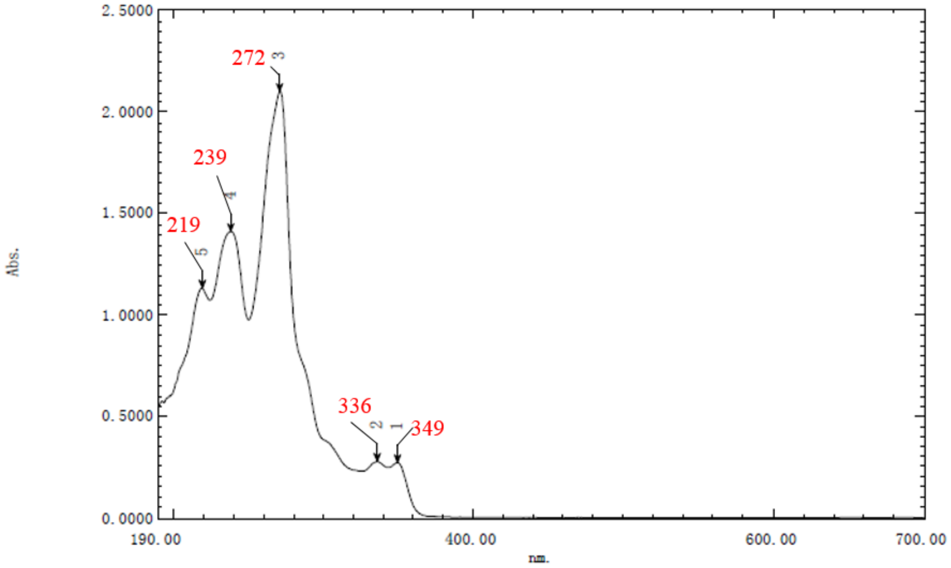

Figure 28. UV spectrum of compound **3.**

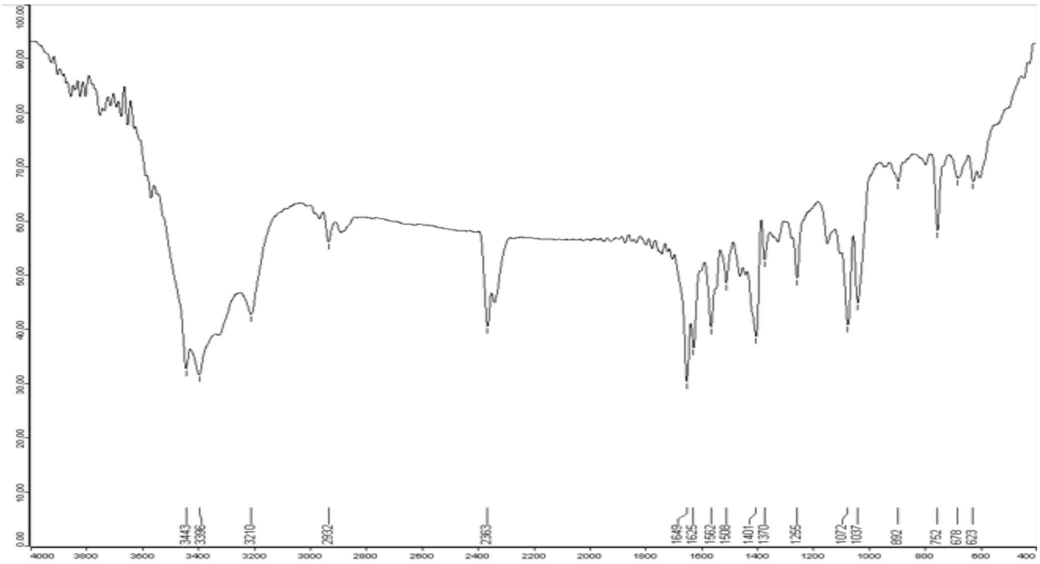

Figure 29. IR spectrum of compound **3.**

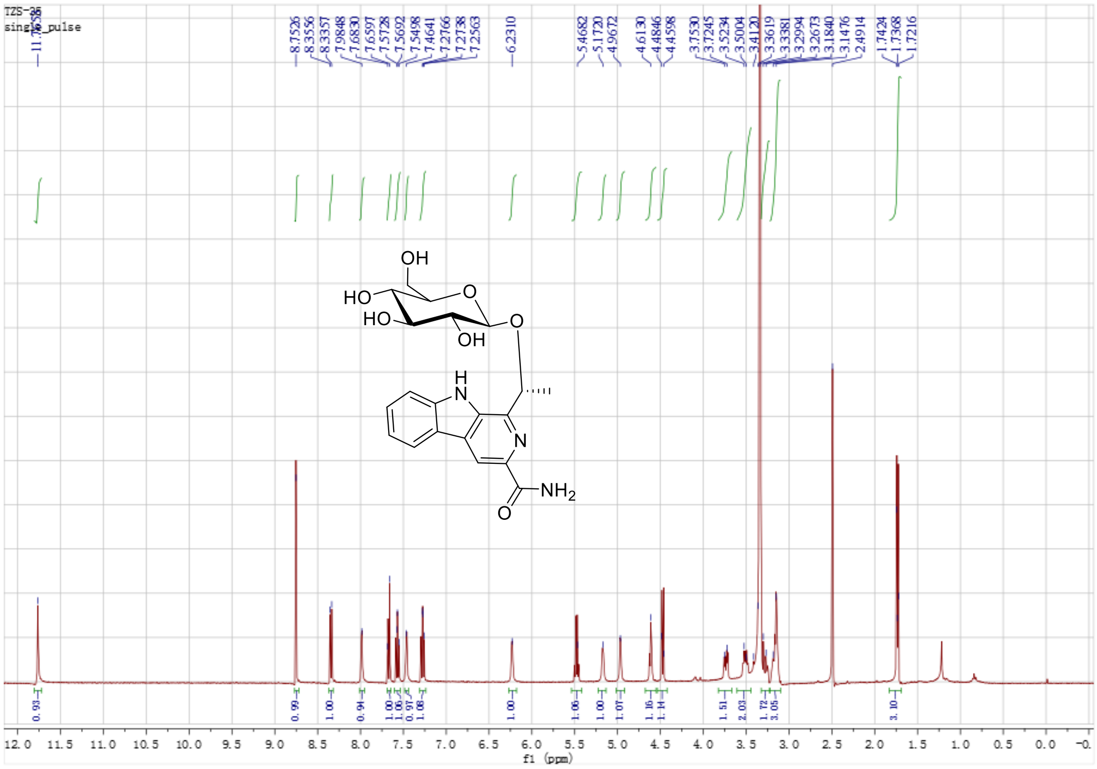

Figure S30. ^{1}H-NMR spectrum of compound **3** (DMSO-d_6, 400 MHz).

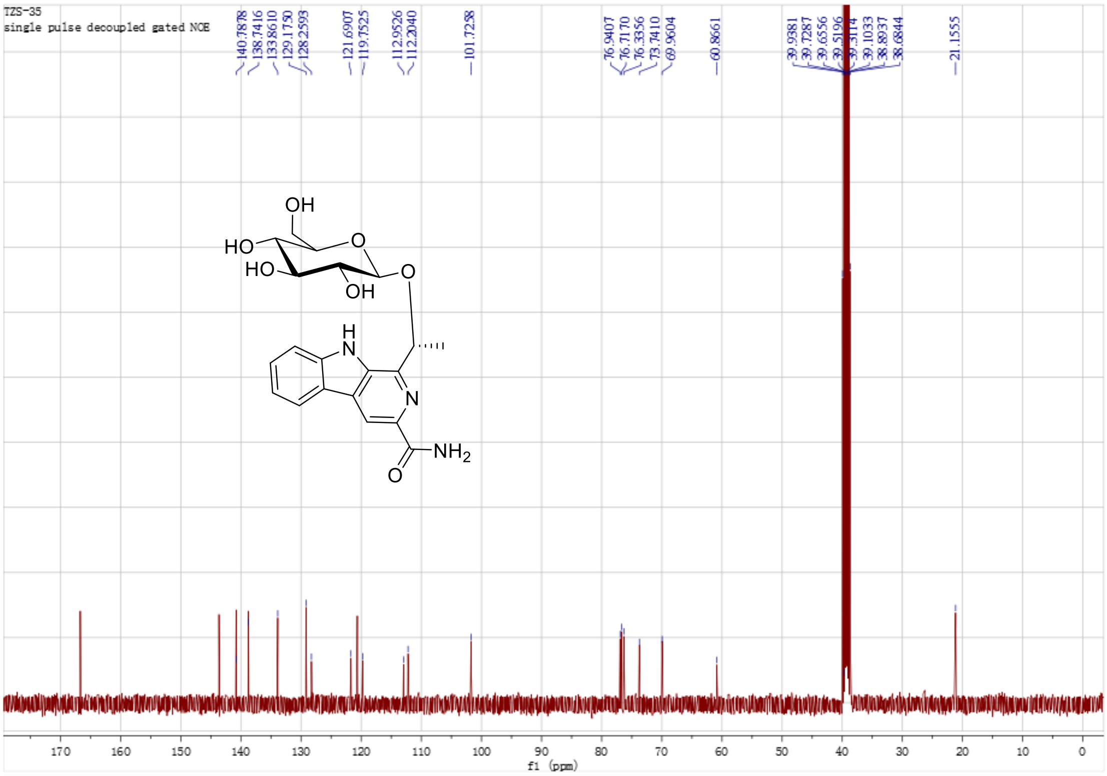

Figure S31. ^{13}C-NMR spectrum of compound **3** (DMSO-d_6, 100 MHz).

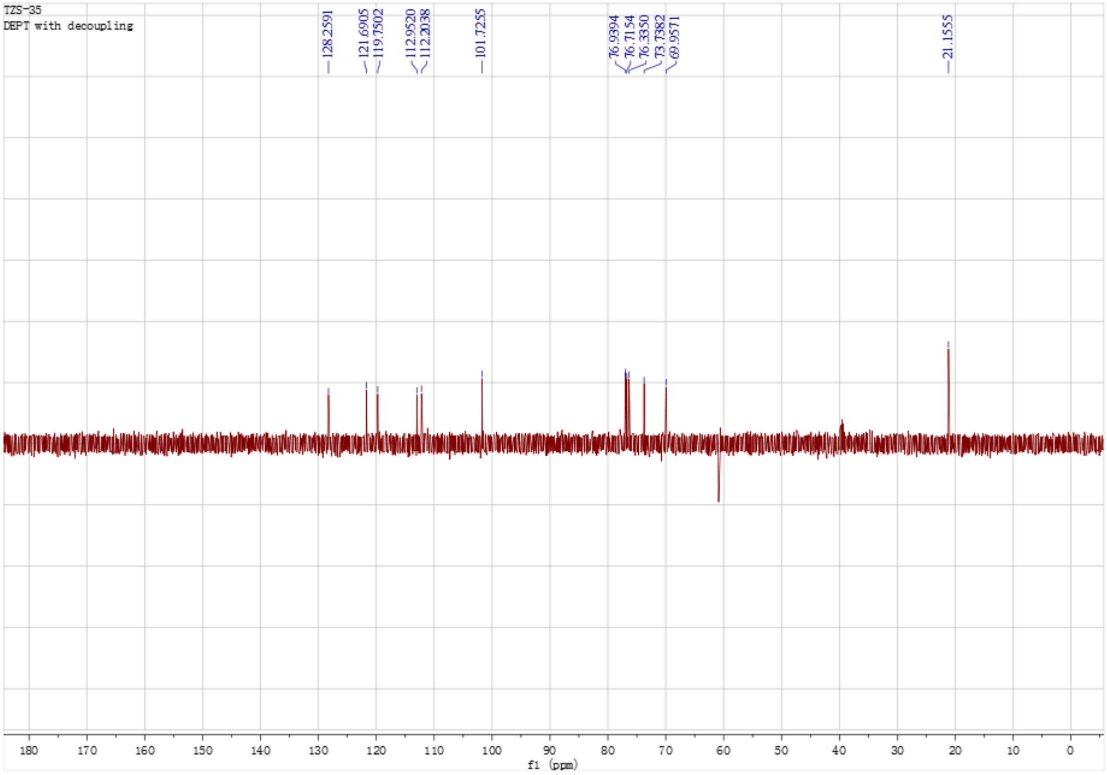

Figure S32. DEPT (135°) spectrum of compound **3** (DMSO-d_6, 100 MHz).

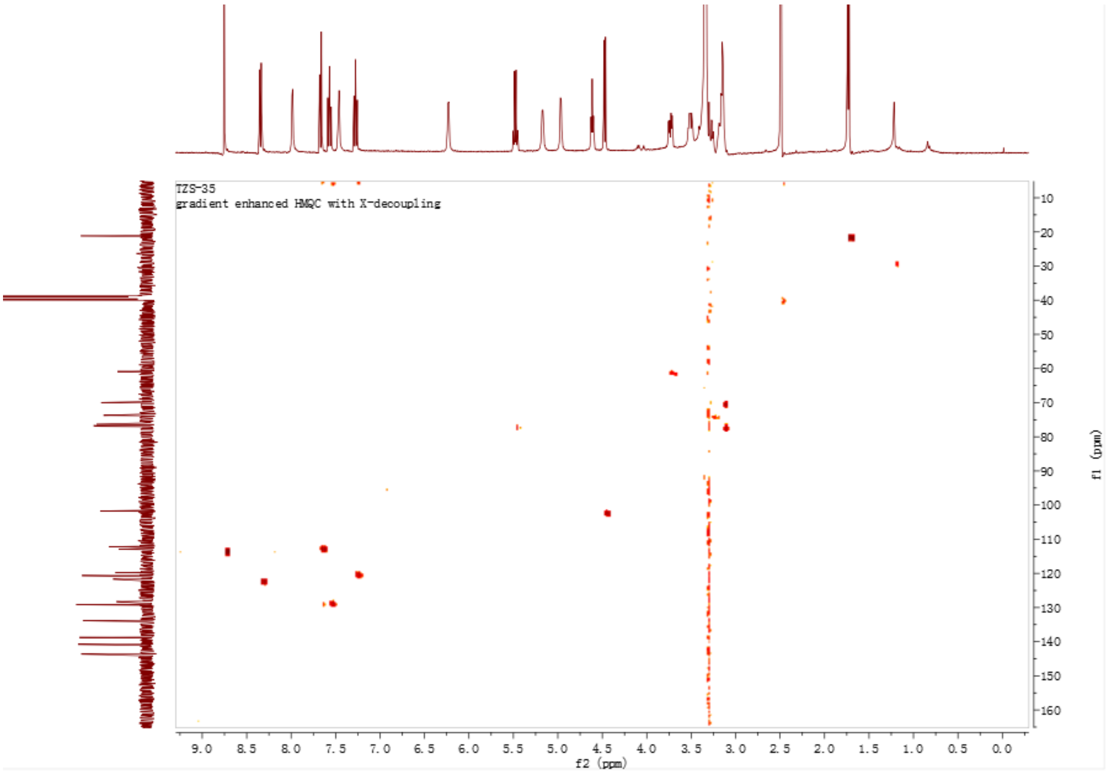

Figure S33. HSQC spectrum of **3** (DMSO-d_6, 400 MHz).

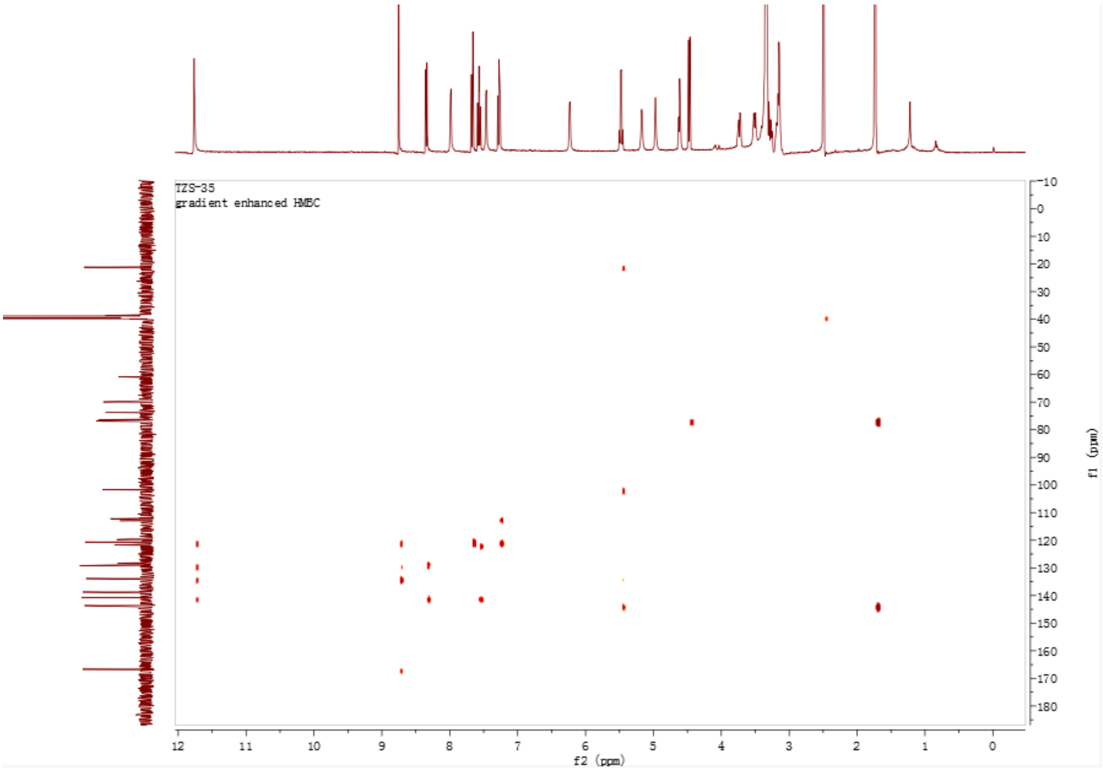

Figure S34. HMBC spectrum of **3** (DMSO-d_6, 400 MHz).

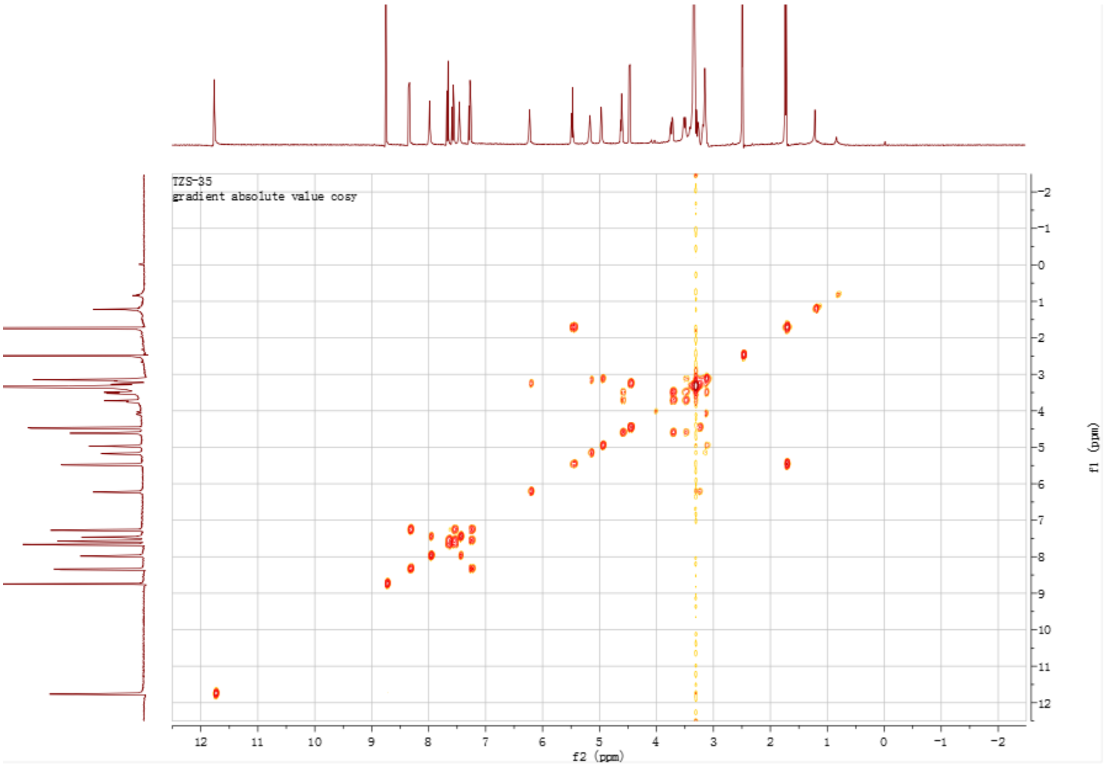

Figure S35. ^{1}H-^{1}H COSY spectrum of **3** (DMSO-d_6, 400 MHz).

References

50 *Spartan 18*; Wavefunction Inc.: Irvine, CA.

51 Gaussian 09, Frisch, M. J.; Trucks, G. W.; Schlegel, H. B.; Scuseria, G. E.; Robb, M. A.; Cheeseman, J. R.; Scalmani, G.; Barone, V.; Mennucci, B.; Petersson, G. A.; et al.Gaussian, Inc., Wallingford CT, 2009.

52 Stephens, P. J.; Harada, N. ECD cotton effect approximated by the Gaussian curve and other methods. *Chirality* **2010**, *22*, 229–233.
